# Species delimitation of the *Dermacentor* ticks based on phylogenetic clustering and niche modeling

**DOI:** 10.7717/peerj.6911

**Published:** 2019-05-10

**Authors:** Fang Wang, Duo Wang, Ge Guo, Yonghong Hu, Jiufeng Wei, Jingze Liu

**Affiliations:** 1 Hebei Key Laboratory of Animal Physiology, Biochemistry and Molecular Biology, College of Life Sciences, Hebei Normal University, Shijiazhuang, Hebei, P.R. China; 2 College of Agriculture, Shanxi Agricultural University, Taigu, Shanxi, P.R. China

**Keywords:** *Dermacentor* ticks, DNA barcoding, Climate niche, Niche overlap, Niche comparisons

## Abstract

Three species belonging to the genus *Dermacentor* (Acari: Ixodidae), *D. marginatus*, *D. nuttalli* and * D. silvarum* are well known as vectors for a great variety of infection pathogens. All three of them are host ticks, which are very similar in morphology characteristics, life cycle, seasonal variation and ecological conditions, making it difficult to distinguish the three species. In the present study, these three species were delimitated based on molecular data and ecological niche. The molecular analysis showed that the three species can be distinguished by COI and ITS2 sequences. We created future potential distribution maps for the three species under climate changes with MaxEnt, which highlighted the different levels of the suitable habitats for each tick species. In addition, niche comparisons among the three species in *Dermacentor* were conducted, and the analysis suggested that niche overlap was relatively high with *D. nuttalli* and *D. silvarum* compared to the other species pairs, which was consistent with the molecular data. Niche equivalency and similarity test confirmed that these *Dermacentor* species were closely related but distinct species. In conclusion, delimitation of these three species within *Dermacentor* was supported by molecular phylogeny and quantitative ecological space. This study will provide deep insights into the biology, ecology, and diversification processes within *Dermacentor* species, and for the development of effective control for ticks.

## Introduction

Ticks are obligate blood-sucking ectoparasites of a great variety of vertebrates, and are considered to be the second most important vectors of pathogens (Dantas-Torres, 2010). They transmit a diverse group of pathogens, including viruses, rickettsia, bacteria, spirochetes and protozoa, which can cause severe damage to humans and domestic animals (Teng & Jiang, 1991; Hajdušek et al., 2013).

The hard tick genus *Dermacentor* contains 36 valid species worldwide with 16 species found in China (Chen et al., 2010; Guglielmone & Nava, 2014; Sun, Zheng & Xu, 2017). Among them, *D*. *silvarum* and *D. nuttalli* are widely distributed in northern China, Russia and Mongolia (Teng & Jiang, 1991; Chen et al., 2010), and *D. marginatus* is mainly in Asia, Europe, and North Africa (Teng & Jiang, 1991; Rubel et al., 2015; Sun, Zheng & Xu, 2017). The three *Dermacentor* species are host ticks, which are very similar in morphology characteristics, life cycle, seasonal variation, host range and ecological conditions, causing difficulties or inaccuracies in morphological identification (Teng & Jiang, 1991; Bayin & Xu, 2001). Accurate identification of species is the basis of the scientific research of ticks, especially important for the effective control of ticks. The morphological information has been the main identification method for ticks in the past; however, this has serious shortcomings, as there are strong overlapping morphological features among these species. In particular, it is difficult to identify these species when the specimens are physically damaged, engorged with blood, at immature stages, even for sibling or cryptic species (Moshaverinia et al., 2009; Lv et al., 2014a). As such, research focused on ultra-morphology and molecular biology is necessary to identify the tick species correctly, and consequently obtain fundamental research for tick control (Zahler, Gothe & Rinder, 1995; Fukunaga et al., 2000; Bayin & Xu, 2001; Chitimia et al., 2009; Chantel, Shaun & Neil, 2010; Lv et al., 2014a; Lv et al., 2014b; Wu et al., 2016; Guo et al., 2016; Chao et al., 2017). According to the study of Lv et al. (2014a), four molecular markers (COI, ITS2, 16S rDNA, 12S rDNA) are suitable for identifying species based on DNA sequences, and COI is the first choice for tick species identification. Thus, we used cytochrome oxidase subunit I (COI) and internal transcribed spacer 2 (ITS2) to identify species in the genus *Dermacentor*.

The role of ecological niches in speciation diversification is of central interest to biologists. Hence, more studies paid attention to the interplay between extrinsic factors and intrinsic organismal traits (Soberón, 2007; Wellenreuther, Larson & Svensson, 2012; Hu et al., 2016; García-Navas & Westerman, 2018), which may influence the geographic range limits of species and even promote lineage diversification (Wellenreuther, Larson & Svensson, 2012; Hu et al., 2016). Among extrinsic factors, climate change is an important factor that affects the species distribution range, physiological characters of insect life history and population dynamics (Hellmann et al., 2007; Bertelsmeier, Guénard & Courchamp, 2013; Bellard et al., 2013; Yousuf et al., 2014). For ticks, most of their life cycle is spent in the environment and all life cycle stages are dependant on climate variables. Therefore climate suitability influences the fitness of tick populations in different regions (Estrada-Peña, 2008). The climate niche-based models have been used extensively to predict species distribution for a wide range of biota, and quantifying niche differentiation among close related taxa is critical for understanding the evolutionary dynamics of animals (Pearman et al., 2008; Wiens et al., 2010; Hu et al., 2016). The quantitative results can be addressed using null models when testing niche comparisons, which will provide more ecology information for close taxa (Warren, Glor & Turelli, 2008; McCormack, Zellmer & Knowles, 2010).

In the present study, we aimed to identify the three *Dermacentor* species using molecular data and ecological niche. Moreover, we predicted the potential distribution of these species around the world, and conducted climatic niche comparisons among the three species. Combined with the phylogenetic relationships, these results will provide further insights into the tick biology, tick-control, and possible diversification processes within *Dermacentor* species.

## Materials & Methods

### Sample collection

*D. silvarum* and *Haemaphysalis longicornis* were collected from Xiaowutai National Natural Reserve of Hebei Province, China. *D. nuttalli* were collected from a farm in the suburb of Yanbian Korean Autonomous Prefecture, Jilin Province, China. All ticks were first identified morphologically under a light microscope, and then verified by molecular data.

### DNA extraction, PCR and sequence analysis

Total genomic DNA was extracted from ticks using the DNeasy blood and tissue Kit (Bioteke, Beijing, China) following the manufacturer’s protocols. Amplification of COI and ITS2 were performed using the respective primer pairs: LCOI490F (5′- GGTCAACAAATCATAAAGATATTGG-3′)/HCO2198R(5′-TAAACTTCAGGGTGA- CCAAAAAATCA-3′) (Folmer et al., 1994; and F3/1 (5′-GGGTCGATGAAGAACGCA- GCCAGC-3′)/R1/1 (5′-TTCAGGGGGTTGTCTCGCCTGATG-3′) (Kulakova et al., 2014). The PCR reactions contained a total of 40 µl consisting of 20 µl 2 ×Taq PCR Master Mix (Biomed, Beijing, China), 17 µl distilled water, 1 µl of each primer and 1 µl DNA template, and a negative control was included for all reactions. The amplifications were conducted on the ProFlex™ PCR System (Thermo Fisher, Waltham, MA, USA). The cycling conditions for COI was as follows: 94 °C for 5 min, 35 cycles of 94 °C for 30 s, 43 °C for 50 s, 68 °C for 1 min, and an extension of 68 °C for 10 min. The protocols for ITS2 was: 95 °C for 10 min, 30 cycles of 94 °C for 30 s, 58 °C for 30 s, 72 °C for 1 min, and an extension of 72° C for 10 min. The PCR products were visualized on 1% agarose, and the most intense products were sent for sequencing in Sangon Biotech (Shanghai, China). Moreover, sequences of COI and ITS2 of portion ticks were obtained from GenBank (http://www.ncbi.nlm.nih.gov/genbank/).

All the sequences were analyzed using Mega 7 (Kumar, Stecher & Tamura, 2016) and phylogenetic analyses were conducted using MrBayes v3.1.2 (Ronquist & Huelsenbeck, 2003). The HKY+I and GTR model were respectively selected for COI and ITS2 data using MrModelTest 2.3 (Nylander, 2004). For the Bayesian analyses, two independent runs were performed for 5,000,000 generations by sampling one tree per 1000 generations. The first 25% of trees were discarded from analysis as burn-in. *H. longicornis* (Ixodidae) was chosen as the outgroup.

### Digital occurrence records

The distribution information of ticks was collated from the Global Biodiversity Information Facility (GBIF) database (https://www.gbif.org/) and Vectormap (http://vectormap.si.edu/index.htm). Further data was obtained from the related published literature (Table S1) and from the georeferenced specimens in the Hebei key laboratory of Animal Physiology, Biochemistry and Molecular Biology, China. Online gazetteers and Google Earth were used to reconstruct the geo-coordinates for each chosen datapoint. Occurrence records of each species were double-checked by DIVA-GIS software (version 5.2) to detect possible errors in georeferencing. A total of 685 occurrence records for *Dermacentor* species were assembled in our analysis (Table S2). Occurrence records are often biased due to the different densities of species distribution. Thus, in order to reduce the sampling bias and remove the spatial autocorrelation, we used a coarse resolution of 5 arc-minute and then randomly selected a single point in each grid cell (Rodríguez-Castaneda et al., 2012; Li, Du & Guo, 2015).

### Climate variables

To characterize the climate heterogeneity across the distribution range for the tick species, we initially compiled all 19 bio-climatic variables, which were download from the Worldclim Global Climate Database (http://www.worldclim.org) (Hijmans et al., 2005), representing minima, maxima and average values of monthly, quarterly, and annual ambient temperature as well as precipitation recorded between 1950 and 2000. Moreover, a spatial resolution of 2.5 arc min (approx. ∼5 km resolution at the equator) of these climate data was used.

The accuracy of a model can be affected by the strong covariance among environmental variables. In order to minimize the multicollinearity among predictor variables, we used principal component analysis (PCA) of all 19 climatic variables in SPSS statistics to identify and remove highly correlated variables (—r— > 0.80) from our models (Table S3). Finally, six climatic environmental variables were selected for the tick species: Bio2 (Mean Diurnal Range), Bio8 (Mean Temperature of Wettest Quarter), Bio12 (Annual Precipitation), Bio15 (Precipitation Seasonality), Bio18 (Precipitation of Warmest Quarter) and Bio19 (Precipitation of Coldest Quarter).

### Climatic niche modeling

In the last few decades, species distribution models (SDMs) became a popular tool for estimating the potential distribution for animals (Park et al., 2017; Wei et al., 2018) and plants (Thuiller et al., 2005; Yi et al., 2016). Lots of SDMs were designed to predict potential distribution of a species, such as MaxEnt, GARP and BIOCLIM (Beaumont, Hughes & Poulsen, 2005). However, the MaxEnt software (version 3.3.3k, http://www.cs.princeton.edu/sc˜hapire/maxent/) has been used mostly frequently to simulate species potential distribution, since it only requires presence-only data (Radosavljevic & Anderson, 2013; Cooper et al., 2016). Many studies had shown that MaxEnt performed well regardless of the number or geographical extent of species records, and outperformed than other methods when predicting the potential distribution of species, especially for the small individual samples (Hernandez et al., 2006; Elith et al., 2011; Bosso et al., 2016; Sultana et al., 2017).

Some recent studies had demonstrated the importance of MaxEnt model settings to balance the performance and complexity of the model, as the default settings of MaxEnt could result in overfit models (Radosavljevic & Anderson, 2013; Merow et al., 2014; Warren et al., 2014). Thus, to avoid overfitting, simultaneously maximize the predictive power and provide the best model for the species, we adopted the R package (version 3.4.2, R Core Team, 2017) ENMeval to select the optimal combination of two important MaxEnt parameters, the value of the regularization multiplier and the combination of feature classes (Muscarella et al., 2014; Naveda-Rodríguez et al., 2016). The “checkerboard2” approach was employed to calculate the standardized Akaike Information Criterion coefficient (AICc). The parameterizations that resulted in the model with the lowest delta AICc score were selected to run the final MaxEnt models (Warren & Seifert, 2011; Radosavljevic & Anderson, 2013). As AICc can only choose the ‘best’ one from a set of models, and can not directly assess the model performance, we inspected the omission rate and tested AUC (area under the curve) of the models that were selected as optimal. Moreover, the parameter for regularization multiplier and feature were set based on the ENMeval analysis. The regularization multiplier was varied from 0.5 to 4 in increments of 0.5, and the following five feature classes were tested: (1) Linear (L); (2) Linear (L) and Quadratic (Q); (3) Linear (L), Quadratic (Q) and Hinge (H); (4) Linear (L), Quadratic (Q), Hinge (H) and Product (P); (5) Linear (L), Quadratic(Q), Hinge (H), Product (P) and Threshold (T).

A minimum convex polygon that was comprising all records of each species were randomly chosen to define background points (Phillips, 2008). Additionally, the logistic output of MaxEnt was used for all analyses. The LPT (lowest presence threshold) was used to define the suitable and unsuitable habitats for all three species. This threshold was a conservative value that was widely used in species distribution modeling, especially when data was collected by different observers and methods over a long period of time (Bosso et al., 2016). The final model was converted into a binary presence/absence map, which was created using the reclassify module from ArcGIS 10.2 (ESRI) by applying this threshold. To maximize the predictive information and simplification of future analysis, the suitable habitat areas for the three tick species were classified into four levels.

### Modeling evaluation

The AUC of the receiver operating characteristic (ROC) (Fielding & Bell, 1997) were used to estimate the performance of the model. The AUC value was ranged from 0 to 1, where a value below 0.5 was interpreted as a random prediction; 0.5–0.7 indicated poor model performance; 0.7–0.9 indicated moderate performance; and a value above 0.9 was considered to have ‘good’ discrimination abilities (Peterson et al., 2011). A 10-fold cross-validation was used to run MaxEnt to prevent random errors from affecting the selection of the validation and prediction samples. To assess the influence of environmental variables on species, the jackknife test was adopted to measure the importance and the percent contributions of each variable.

### Niche overlap test

Niche overlaps among species were quantified using the PCA-env method proposed by Broennimann et al. (2012). PCA was used to transform the environmental space of the investigative environmental variables into a two-dimensional space defined by the first two principal components (Strubbe et al., 2013; Strubbe, Beauchard & Matthysen, 2015). Then, the two-dimensional environmental space was projected onto a 100 ×100 PCA grid of cells bounded by the maximum and minimum PCA values in the background data. A kernel function was used to smooth the climatic space defined in the gridded PCA climatic spaces based on the first two principal components (PCs) (Petitpierre et al., 2012). The niche overlap among the species was measured directly by Schoener’s D from the ecological niche space (Warren, Glor & Turelli, 2008). The Schoener’s D was an index which varied from 0 (no overlap) to 1 (overlap). Additionally, niche equivalency and a similarity test were also implemented and if the niche overlap value fell outside the 95% confidence interval of the null hypotheses, equivalency of the two niches was rejected. And in the similarity test, a *p* value >0.05 indicated that the niches were no more similar than expected by chance.

All GIS analyses were performed using ArcGIS version 10.2 (ESRI). The “ecospat” package in R was used to implement this analysis (Broennimann, Di Cola & Guisan, 2018).

## Results

### Phylogenetic divergence

**Figure 1:**
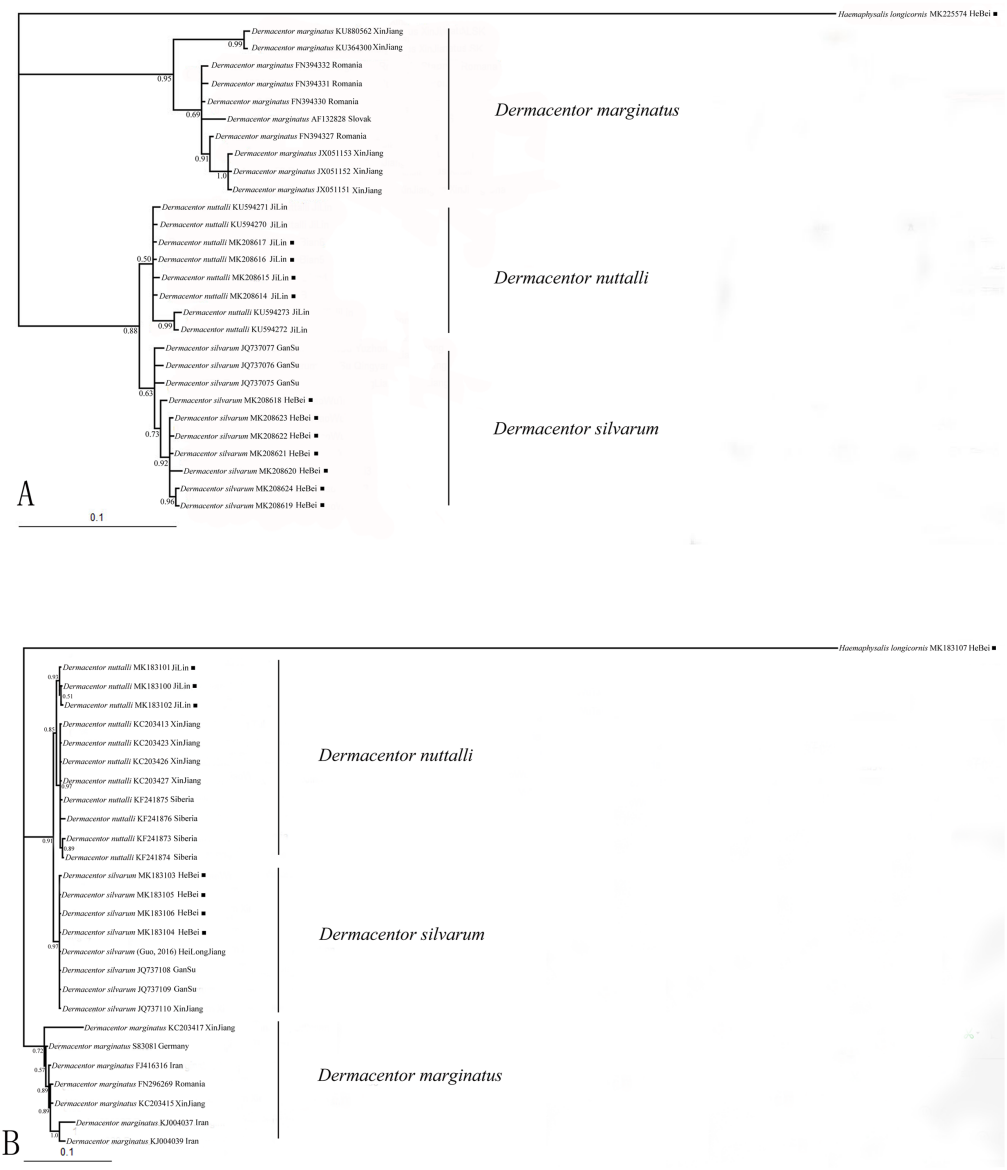
(A) The bayesian inference (BI) analysis based on COI sequences of the three species in *Dermacentor*; (B) The bayesian inference (BI) analysis based on ITS2 sequences of the three species in *Dermacentor* Numbers on the nodes are posterior probability (PP); ■ sequences amplified from our specimens.

Nucleotide sequences obtained in this study were used to assess the phylogenetic relationship of the tick species. The length of the COI sequences were 625 bp after edge trimming. ITS2 sequences were ranged from 857 bp in *H. longicornis* to 1039 bp in *Dermacentor* species. Two phylogenetic trees (based on COI and ITS2) obtained from Bayesian analyses showed similar topology (Figs. 1A, 1B). All obtained sequences clustered tightly in a branch and formed a distinct lineage. Moreover, the two species *D. silvarum* and *D. nuttalli* grouped together, and *D. marginatus* was in a separate cluster in the two analyses.

### ENMeval data and model performance for potential distribution

The results of ENMeval were shown in Table S4 and Fig. S1. Regularization multiplier = 1, feature combinations = Linear, Quadratic, Hinge, Product and Threshold (LQHPT) were chosen to MaxEnt configuration for *D. marginatus*; regularization multiplier = 0.5, feature combinations = Linear and Quadratic (LQ) were chosen as the optimal configuration for *D. nuttalli* and *D. silvarum*.

Based on 10-fold cross validation, the model performance for the three species were all better than random, with a mean AUC value of 0.948 ±  0.013 for *D. marginatus*, 0.952 ±  0.019 for *D. nuttalli* and 0.940 ±  0.044 for *D. silvarum*. These results indicated that the model performed well in predicting the species distribution for all three species. A threshold value of 0.0021 for *D. marginatus*, 0.0034 for *D. nuttalli* and 0.0037 for *D. silvarum*, which were obtained from the LPT analysis. The suitable habitat area for these species were classified into four levels: <threshold indicated unsuitable habitat; threshold-0.4 indicated low habitat suitability; 0.4–0.6 indicated moderate habitat suitability; and >0.6 indicated high habitat suitability.

### Important environmental variables for ticks

**Figure 2:**
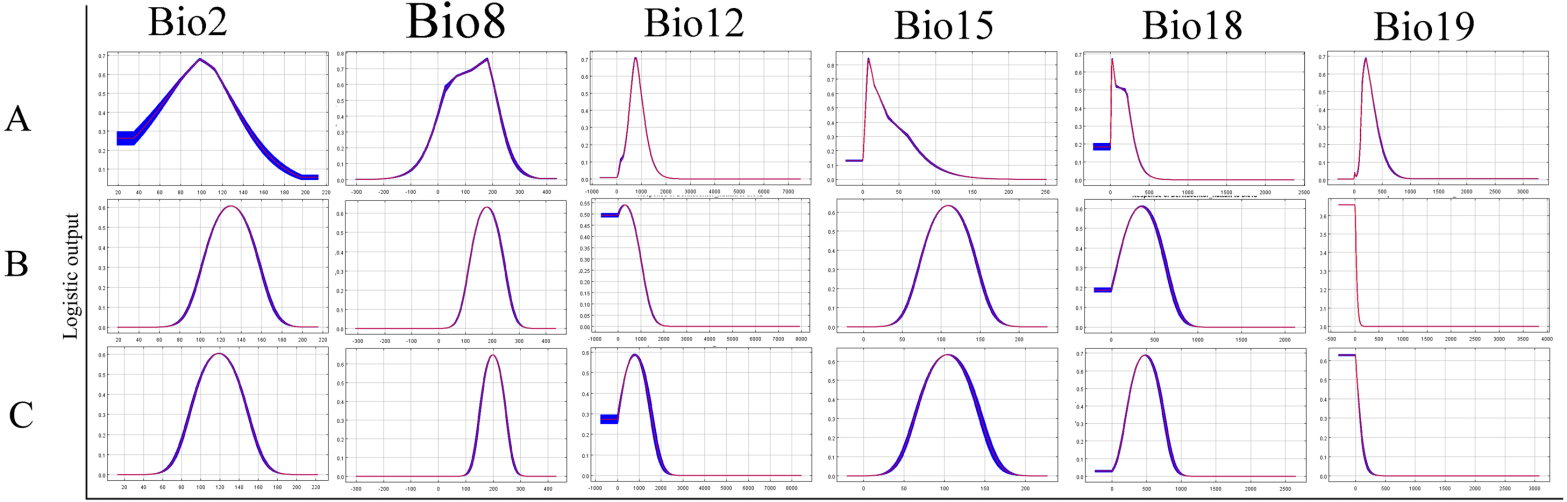
Response curves showing the relationships between the probability of presence of *Dermacentor* ticks and six bioclimatic variables respectively. (A) *Dermacentor marginatus*; (B) *D. nuttalli*; (C) *D. silvarum.*

**Figure 3:**
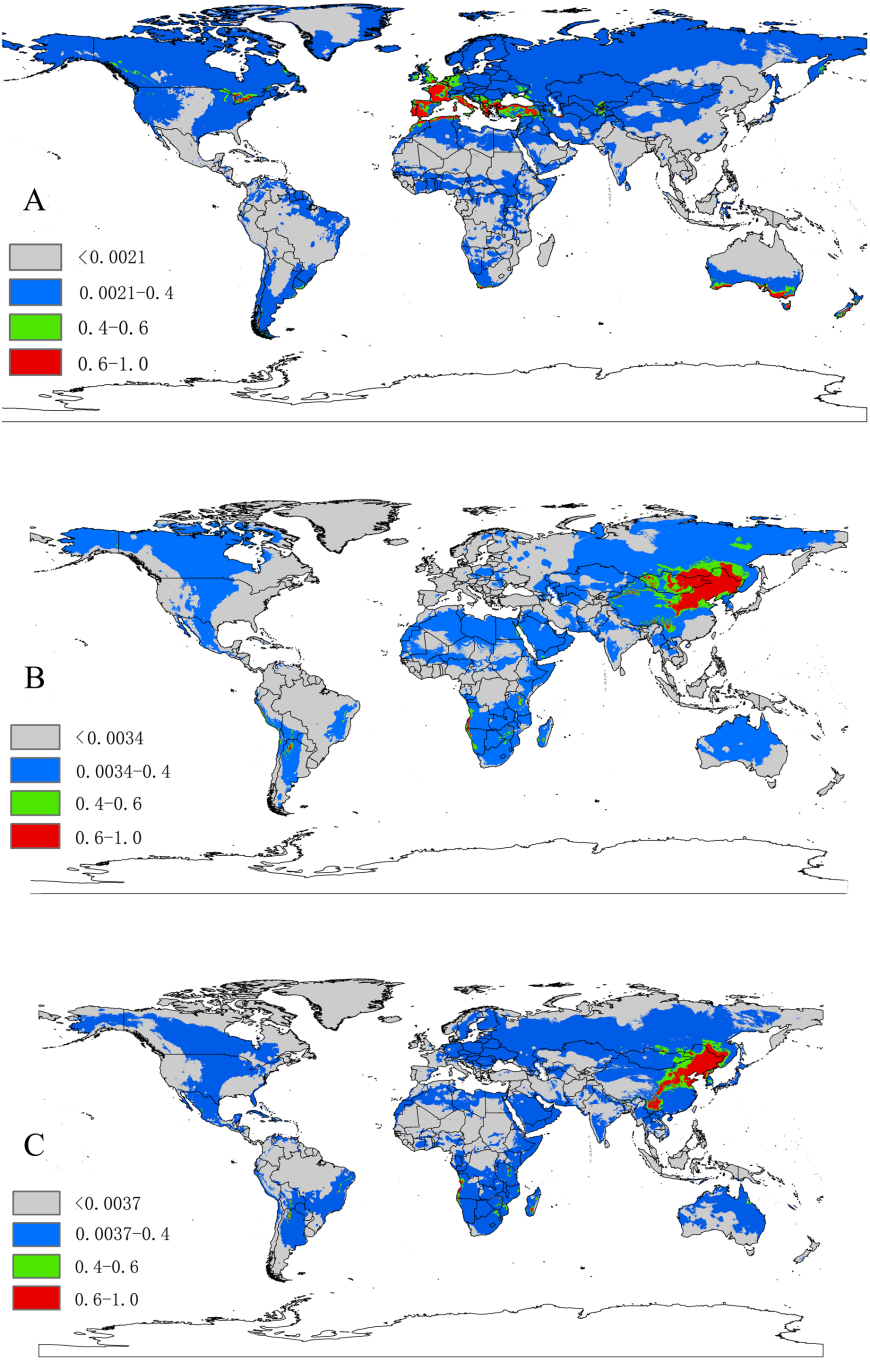
(A) The potential distribution map for *Dermacentor marginatus*; (B) The potential distribution map for *D. nuttalli*; (C) The potential distribution map for *D. silvarum*. Red, highly suitable areas; Green, Moderately suitable areas; Blue, Low suitable areas; Gray, Unsuitable areas. The base maps were created with Natural Earth Dataset (http://www.naturalearthdata.com/).

The relative contributions of the six environmental variables for the three species were shown in Table 1 and Fig. 2. Precipitation of coldest quarter (Bio19) and Mean temperature of wettest quarter (Bio8) had the largest contributions to the distribution model for *D. marginatus*. These two factors explained 73.3% of the modeled distribution. The distribution of *D. nuttalli* was mainly constrained by precipitation seasonality (Bio15) and Mean temperature of wettest quarter (Bio8), which explained 54.6% of the distribution. *D. silvarum* was mainly constrained by Precipitation of warmest quarter (Bio18) and Mean temperature of wettest quarter (Bio8), which accounted for 53% of the modeled distribution. Mean temperature of wettest quarter (Bio8) was the second highest contribution for the model distribution in all three species. Among the six environmental variables that our analysis focused on, precipitation conditions were more important than other factors when creating the distribution map for these species. The potential distribution of these species were shown in Fig. 3 and Fig. S2. The potential distribution maps predict that *D. marginatus* would be found mainly in European, such as France, Italy, Spain, Greece and Portugal, which have highly suitable climate for this species. However, our analysis predicted that *D. nuttalli* and *D. silvarum* would have similar distribution area which mainly focus on eastern of Eurasia. Moreover, compared to *D. silvarum*, *D. nuttalli* had wider potential distribution, including Mongolia and northern China.

**Table 1:** Percentage of variable contribution to the model construction. For each species taxon, the two variables with highest contributions are presented in bold.

Variable	*D. marginatus*	*D. nuttalli*	*D. silvarum*
Mean diurnal range (Bio2)	4.8%	1.9%	4.6%
Mean temperature of wettest quarter (Bio8)	**23.7**%	**23.6**%	**24**%
Annual precipitation (Bio12)	2.2%	12.8%	16.1%
Precipitation seasonality (Bio15)	4.9%	**31**%	18.3%
Precipitation of warmest quarter (Bio18)	14.8%	10.3%	**29**%
Precipitation of coldest quarter (Bio19)	**49.6**%	20.5%	8%

### Niche comparisons

The ordination approach using PCA-env revealed the niche patterns for each species pair in the environment space (Table 2 and Fig. S3). The PCA-env resulted in the species pair *D. marginatus* and *D. nuttalli* showed two PCA axes explaining 71.98% of all climatic variables, and two PCA axes explaining 73.24% for species pair *D. marginatus* and *D. silvarum*. The analysis also revealed that 74.28% of all climatic variables for *D. nuttalli* and *D. silvarum* were explained by two PCA axes. Furthermore, niche overlaps showed a great variability in the E-space inhabited by the different species. There were 17% niche overlap between *D. nuttalli* and *D. silvarum* (Schoener’s D = 0.17), 9% between *D. marginatus* and *D. nuttalli* (Schoener’s D = 0.091), and only 2% between *D. marginatus* and *D. silvarum* (Schoener’s D = 0.026). Of the three species that were analyzed, niche overlaps of *D. nuttalli* and *D. silvarum* were highest.

**Table 2:** Niche comparisons for the three species in *Dermacentor*. Niche overlap values are presented for the comparisons of niche similarity and equivalency of species 1 with species 2. All of the comparisons between the species highlight the nonequivalency of their niche.

Species pairs		Niche overlap (Schoener’s D)	Niche similarity	Niche equivalency
1	2		1 →2	1 →2
*D.marginatus*	*D.nuttalli*	0.091	ns	Different*
*D.nuttalli*	*D.silvarum*	0.170	ns	Different
*D.marginatus*	*D.silvarum*	0.026	ns	Different

**Notes.**

ns, no significantly different.

The environmental space occupied by each species as determined by PCA-env were also shown in Fig. S3 and Table 2. The null hypothesis of the niche equivalency test was rejected for all possible pairwise comparisons, suggesting that climate niche among these species pairs were significantly distinct. On the other hand, in niche similarity analysis, null hypothesis testing were held on all species pairs. For the *Dermacentor* species investigated, the niche similarities were higher than expected by chance. Taken together, we found that the niche of these three species were similar but not identical, highlighting that while these three species are closely related, they represent distinct species.

## Discussion

Together with morphological and genetic traits, climatic variables have recently been considered to be an important component of species delimitations (Raxworthy et al., 2007; Cornetti et al., 2015). In this study, we took a multidisciplinary approach by conducting genetics and ecological niche analyses to identify species boundaries of three *Dermacentor* species.

DNA barcoding has become a popular tool for species delimitation in ticks, which makes the ticks identification accurately and rapidly (Chitimia et al., 2009; Chantel, Shaun & Neil, 2010; Lv et al., 2014a; Lv et al., 2014b; Wu et al., 2016). Here, COI and ITS2 sequences were used to identify *Dermacentor* species. The Bayesian analysis of the ticks, molecular data showed the phylogenetic relationships of the three *Dermacentor* species, and trees made using ITS2 and COI sequences showed consistent topologies (Figs. 1A, 1B). According to the study of Hebert & Barrett (2005), species identification was successful when all sequences from the same species clustered on a single branch and if sequences of different individuals from the same species fell into different branches, it was considered to be unreliable. In our present study, the sequences we obtained from the different species formed three distinct lineages that were consistent with the morphological, indicating that the three species can be distinguished by COI and ITS2 sequences. Thus, COI and ITS2 sequences could provide useful genetic markers for the specific identification and genetic characterization of ticks in *Dermacentor*. The phylogentic analysis suggested that *D. silvarum* and *D. nuttalli* were more closely related, and this was consistent with other molecular data and morphological characteristics (Bayin & Xu, 2001; Kulakova et al., 2014; Sun, Zheng & Xu, 2017). Meanwhile, *D. marginatus* formed a separate cluster of branches that was distant from the other two species, consistent with a previous study that showed *D. silvarum* and *D. nuttalli* were more closely related than *D. marginatus* (Kulakova et al., 2014). Moreover, these new sequences will be useful for future identification of these species and promote the phylogenetic research for ticks.

In interpreting the ENMs, it is important to distinguish the known distribution of a species, as indicated by the species records and their potential distribution (Soberón & Peterson, 2005; Ortega-Andrade et al., 2015). In this study, we used MaxEnt to predict the potential distribution of the three *Dermacentor* species based on the climatic environmental variables. MaxEnt produced highly accurate predictions of AUC value, which was greater than 0.9 for all species. As we all known, the three *Dermacentor* species can carry a large variety of tick-borne pathogens including *Babesia*, *Borrelia*, *Rickettsia* and viruses (Battsetseg et al., 2001; Selmi et al., 2008; Tian et al., 2012; Wen et al., 2014; Zhang et al., 2014). Through the transmission of pathogens, these ticks can cause severe diseases to human health, such as Lyme disease, encephalitis, spotted fever, babesiosis, human granulocytic ehrlichiosis (HGE), and tick-borne lymphadenopathy, as well as economic loss to livestock production (Moshaverinia et al., 2009; Kulakova et al., 2014; Yu et al., 2015). The potential distribution map of the ticks will be beneficial in developing strategies to monitor future infestations in currently uninfected regions. Therefore, strict quarantine measures are needed in areas deemed to be highly suitable for each species, in order to make effective barriers to prevent the transmission or local adaption for these species. However, our model only considered the abiotic factors (climate variable), other biotic factors, especially the host animals, that would limit the accuracy of the prediction in our work. Additionally, human activities and international exchanges would promote the occurrence of species, which increase the risk of these species, particularly for the transmission of the pathogens. We suggest that special control measures should be taken to limit the spread of ticks to potentially suitable regions to prevent future infestations.

In the current study, ecological niche modeling and ordination technique were applied to identify the environmental constraints for the distribution of ticks. The climatic parameters provided a combination of means, extremes and seasonal differences in variables known to influence the species distribution (Root et al., 2003). Our models indicated several environmental variables that explain the current distribution of the tick species throughout the world. The results revealed that these three species differ in their realised eological niches, represented here by their modeled climate envelopes. The effect of environmental variables on constraining the distribution of each species varied considerably. *D. marginatus* was mainly constrained by Precipitation of coldest quarter (Bio19) and *D. nuttalli* were mainly shaped by Precipitation seasonality (Bio15). However, *D. silvarum* was mainly constrained by Precipitation of warmest quarter (Bio18). Interestingly, the second most constraining factor was Mean temperature of wettest quarter (Bio8) for all three species.

According to the ecological species concept, individuals occupying the same niche or adaptive zone constitute a species; that is to say, a different lineage has to occupy a different niche (Andersson, 1990; De Queiroz, 2007). Quantifying the niche differences among closely related species was of fundmental interest in eclology and evolutionary biology, as these differences would provide a solid foundation for future studies and provide insights into the mechanisms underlying niche separation at broad-scale geographic patterns (Underwood, Chapman & Connell, 2000; Wellenreuther, Larson & Svensson, 2012). Here we quantified the climatic niches of three closely related congeneric tick species. The results of our modeling showed that these three species did not occupy the same niche. A niche overlap test showed that *D. nuttalli* and *D. silvarum* had a relatively high degree of overlap compared to the other two species pair comparisons. This means that the two species occupied a more similar niche, and the relationship of the two species was closely related, which was consistent with the molecular data in the current study. Niche equivalency test among all pairs suggested a lack of ecological exchangeability and all species occupied different climatic space. However, the niche similarity test showed that these species share more climate niche characteristics than would be randomly expected. Based on these findings, we found that there was a close relationship among these three species, and they shared climatic niche spaces. Our results further confirm that these three species share certain characteristics, but still represent distinct species.

## Conclusions

In summary, we delimitated *D. marginatus*, *D. nuttalli* and *D. silvarum* based on molecular phylogeny and quantitative ecological space analyses. These three species were closely related but different taxa, not only in the terms of molecular phylogenetic analysis, but also from the results of niche overlap analysis. This study will provide important information for future studies on developing effective controls for *Dermacentor* ticks.

##  Supplemental Information

10.7717/peerj.6911/supp-1 Figure S1 The results of ENMeval for* Dermacentor marginatus*, *D. nuttalli* and* D. silvarum*

10.7717/peerj.6911/supp-2 Figure S2 The localities and potential distribution maps for *Dermacentor* ticks*.* (A) *Dermacentor marginatus;* (B) *D. nuttalli;* (C) *D. silvarum* The base maps were created with Natural Earth Dataset ( http://www.naturalearthdata.com/).

10.7717/peerj.6911/supp-3 Figure S3 The ordination approach using PCA-env revealed the niche patterns for each species pairs in environment space by ENMs

10.7717/peerj.6911/supp-4 Table S1 References used to compile the dataset

10.7717/peerj.6911/supp-5 Table S2 The distribution of the locations used in the study

10.7717/peerj.6911/supp-6 Table S3 Correlation analysis of environmental variables

10.7717/peerj.6911/supp-7 Table S4 ENMeval results for *Dermacentor marginatus*, *D. nuttalli* and* D. silvarum* from SDMs

10.7717/peerj.6911/supp-8 Supplemental Information 1 COI sequences obtained from the present work

10.7717/peerj.6911/supp-9 Supplemental Information 2 ITS2 sequences obtained from the present work

## Additional Information and Declarations

### DNA Deposition

The following information was supplied regarding the deposition of DNA sequences: The sequences described in the present work are available in the Supplemental Files.

### Data Availability

The following information was supplied regarding data availability: Date is available at NCBI, accession numbers: MK208614 to MK208624, MK183100 to MK183107, and MK225574.
