# Supplementary figures and images for: Species delimitation of the Dermacentor ticks based on phylogenetic clustering and niche modeling
Source: PeerJ. 2019 May 10;7:e6911. doi: 10.7717/peerj.6911 (PMC6512763; doi:10.7717/peerj.6911)

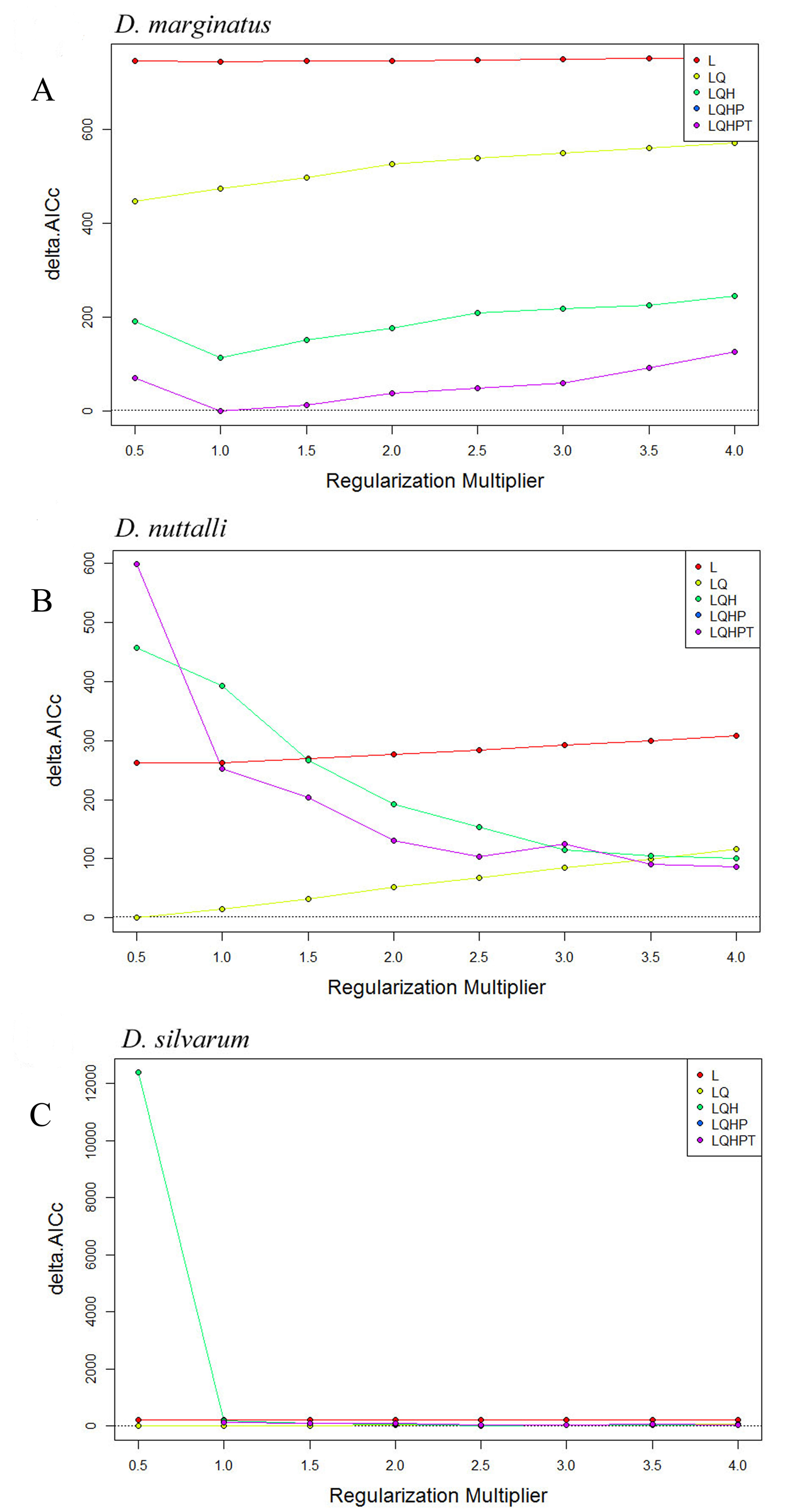

Supplement: Figure S1 [file peerj-07-6911-s001.png]

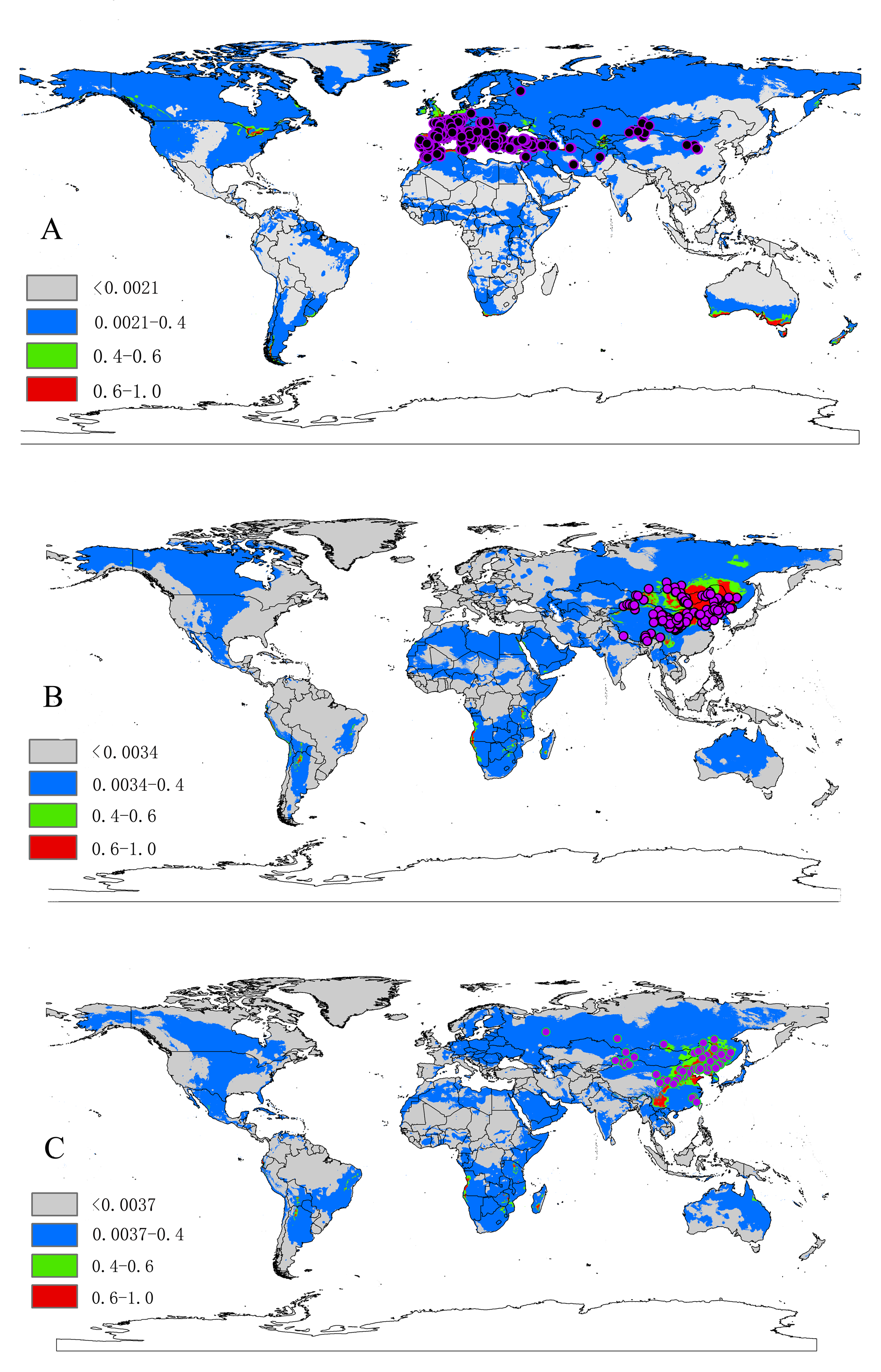

Supplement: Figure S2 — The base maps were created with Natural Earth Dataset ( http://www.naturalearthdata.com/). [file peerj-07-6911-s002.png]

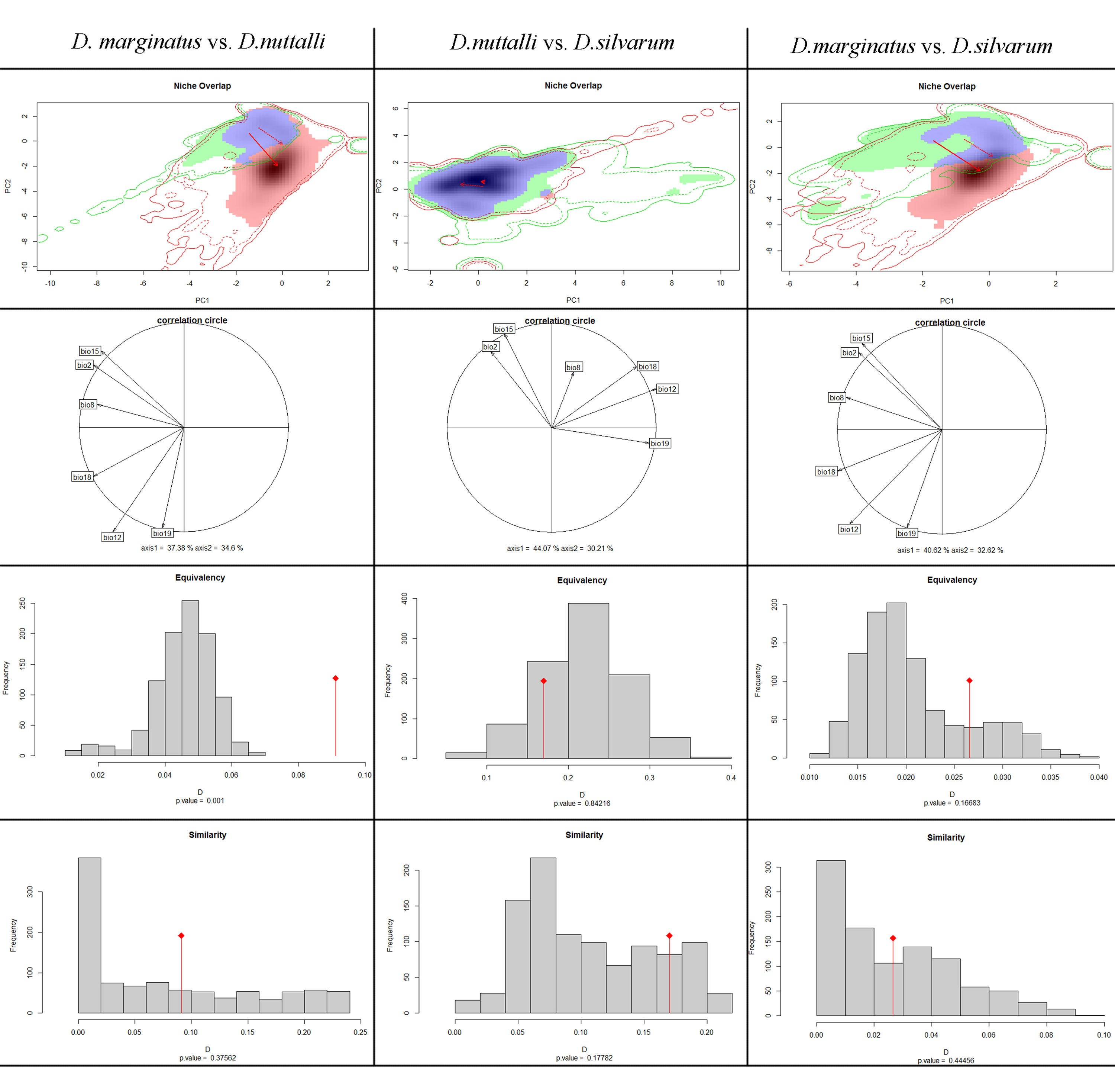

Supplement: Figure S3 [file peerj-07-6911-s003.png]
