# Supplementary material for: Species delimitation of the Dermacentor ticks based on phylogenetic clustering and niche modeling
Source: PeerJ. 2019 May 10;7:e6911. doi: 10.7717/peerj.6911 (PMC6512763; doi:10.7717/peerj.6911)
Supplement: Table S1 [file peerj-07-6911-s004.doc]

Table S1. References used to compile the dataset

Aydin L, [Bakirci](http://xueshu.baidu.com/s?wd=author:(Bakirci S) &tn=SE_baiduxueshu_c1gjeupa&ie=utf-8&sc_f_para=sc_hilight=person) S. 2007. Geographical distribution of ticks in Turkey. *Parasitology Research* 101(S2):S163–S166.

Bayin CH, Xu XZ. 2001. Ultrastructural comparison of *Dermacentor silvarum* and *D. nuttalli*. *Chinese Journal of Veterinary Science and Technology* 31(6):27–30.

[Berthová](http://xueshu.baidu.com/s?wd=author:(BerthovÃ  L) &tn=SE_baiduxueshu_c1gjeupa&ie=utf-8&sc_f_para=sc_hilight=person) L, [Slobodní­K](http://xueshu.baidu.com/s?wd=author:(SlobodnÃ­k V) &tn=SE_baiduxueshu_c1gjeupa&ie=utf-8&sc_f_para=sc_hilight=person)V, [Slobodní­K](http://xueshu.baidu.com/s?wd=author:(SlobodnÃ­k R) &tn=SE_baiduxueshu_c1gjeupa&ie=utf-8&sc_f_para=sc_hilight=person)R, Olekšák M, Sekeyová Z, Svitálková Z, Kazimírová M, Špitalská E. 2016. The natural infection of birds and ticks feeding on birds with *Rickettsia* spp. and *Coxiella burnetii* in Slovakia. *Experimental & Applied Acarology* 68:299–314.

Boldbaatar B, Jiang RR, Fricken ME, Lkhagvatseren S, Nymadawa P, Baigalmaa B, Wang YW, Anderson BD, Jiang JF, Gray GC. 2017. Distribution and molecular characteristics of rickettsiae found in ticks across Central Mongolia. *Parasites & Vectors* 10:61.

Chitimia L, Lin RQ, Cosoroaba I, Wu XY, Song HQ, Yuan ZG, Zhu XQ. 2010. Genetic characterization of ticks from southwestern Romania by sequences of mitochondrial cox1 and nad5 genes. *Experimental & Applied Acarology* 52:305–311.

Chisua V, Foxia C, Mannub R, Sattaa G, Masalaa G. 2018. A ﬁve-year survey of tick species and identification of tick-borne bacteria in Sardinia, Italy. *Ticks and Tick-borne Diseases* 9:678–681.

Guo DH, Zhang Y, Fu X, Gao Y, Liu YT, Qiu JH, Chang QC, Wang CR. 2016. Complete mitochondrial genomes of *Dermacentor silvarum* and comparative analyses with another hard tick *Dermacentor nitens*. *Experimental Parasitology* 169:22–27.

Guo DH. 2016. Differentiation of *Dermacentor silvarum* and *D. nuttalli* by PCR-RFLP and studies complete mitochondrial genome sequences of *D. silvarum*. Thesis, Heilongjiang Bayi Agricultural University.

Hoogstraal H, Valdez R. 1980. Ticks (Ixodoidea) from wild sheep and goats in Iran and medical and veterinary implications. *Fieldiana Zoology* no. 6.

Hoogstraal H. 1971. Biological Patterns in the Afghanistan Tick Fauna. [*Proceedings of the 3rd International Congress of Acarology*](https://link.springer.com/book/10.1007/978-94-010-2709-0) 511–514.

Hua MT, Jin ZQ, Fan YC, Wang ZT, He C. 1999. Investigations on ticks in afforested and livestock breeding area of Haba River TieReKeTi. *Journal of Medical Pest Control* 15(11):563–565.

Huang TP, Zhang JB, Lu JS, Wu M, He HY, GeRi LT. 2017. Identification and gene polymorphism analysis of dominant ticks in Hulun Buir, Inner Mongolia. *Chinese Journal of Veterinary Medicine* 37:2108–2113.

Igolkina Y, Rar V, Vysochina N, Ivanov L, Tikunov A, Pukhovskaya N, Epikhina T, Golovljova I, Tikunova N. 2018. Genetic variability of *Rickettsia* spp. in *Dermacentor* and *Haemaphysalis* ticks from the Russian Far East. *Ticks and Tick-borne Diseases* 9:1594–1603.

Jiang ZJ. 1985. A morphological study of *Dermacentor nuttalli* olenev and *Dermacentor silvarum* olenev. *Acta Entomologica Sinica* 28(1):60–69.

Kazimírová M, Hamšíková Z, Kocianová E, Marini G, Mojšová M, Mahríkova L, Berthová L, Slovák M, Rosá R. 2016. Relative density of host-seeking ticks in different habitat types of south-western Slovakia. *Experimental & Applied Acarology* 69:205–224.

Khasnatinov MA, Liapunov AV, Manzarova EL, Kulakova NV, Petrova IV, Danchinova GA. 2015. The diversity and prevalence of hard ticks attacking human hosts in Eastern Siberia (Russian Federation) with first description of invasion of non-endemic tick species. [*Parasitology Research*](http://www.baidu.com/link?url=l2f1ekYBr6-LljXdIqbS--OEzEXQaTcCnYpLWQRW7CWvmPc0oUYE2u_Q0VwETkEPEJ0gHH6sPLNDHInnvE3Z86ezuhK_2wE38x7M_RTcviZsxpQuhAgHL5si3CKPPlw6ZjGLuAHQ5BPqdSgfo1GEEADHNVaCyPPuxlhfn__hWcm4NKVjgCLsIFI4LDqIhSAAtiyOMhcivVPRDpFts4VEQq3GOa42M6wwNnF53QX72pDzkNW3L8KSxShPoEog2a_ypdPPU2bPFZ798ODmvzs8o_) 115:501–510.

Kotovskii NYu, Bugmyrin SV. 2013. A finding of *Dermacentor marginatus* (Acari, Ixodidae) in Karelia. [*Entomological Review*](https://www.researchgate.net/journal/0013-8738_Entomological_Review) 92:490–491.

Kulakova NV, Khasnatinov MA, Sidorova EA, Adel`shin RV, Belikov SI. 2014. Molecular identification and phylogeny of *Dermacentor nuttalli* (Acari: Ixodidae). [*Parasitology Research*](http://www.baidu.com/link?url=l2f1ekYBr6-LljXdIqbS--OEzEXQaTcCnYpLWQRW7CWvmPc0oUYE2u_Q0VwETkEPEJ0gHH6sPLNDHInnvE3Z86ezuhK_2wE38x7M_RTcviZsxpQuhAgHL5si3CKPPlw6ZjGLuAHQ5BPqdSgfo1GEEADHNVaCyPPuxlhfn__hWcm4NKVjgCLsIFI4LDqIhSAAtiyOMhcivVPRDpFts4VEQq3GOa42M6wwNnF53QX72pDzkNW3L8KSxShPoEog2a_ypdPPU2bPFZ798ODmvzs8o_) 113:1787–1793.

Li WX. 1987. The geographical distribution of ticks in Liaoning Province. *Acta Entomologica Sinica* 30:180–185.

Li ZG, Ma FH, Wu MY, Ma L, Hu X, Ding GJ, Liang XR, Ma HR. 1998. A survey of Ixodoidea distribution in NingXia. *Chinese Journal of Vector Biology and Control* 9:44–45.

Liao HR, Yu X. 1995. Investigation of ticks in Fujian. *Endemic Diseases Bulletin* 10:50–52.

Liu HH. 2017. Survey on ticks species distribution and molecular epidemiology of three associated tick-borne microorganism in Jilin and Heilongjiang Province. Thesis, Jilin Agricultural University.

Liu HH, Li ZY, Wang ZD, He B, Wang SC, Wei F, Tu CC, Liu Q. 2016. The first molecular evidence of severe fever with thrombocytopenia syndrome virus in ticks in Jilin, Northeastern China. *Ticks and Tick-borne Diseases* 7:1280–1283.

Liu MS, Che DC, Huang KJ. 1996. Catalogue of ticks and key of Ixodoidae in Shanxi Department of parasitology. *Journal of Shanxi Teacher’s University Natural Science Edition* 10:35–37.

Liu DX. 2014. Morphological study of two species of ticks in Qinghai Province and epidemiology survey on the prevalence of tick-borne Piroplasmosis. Thesis, Qinghai University.

Lv JZ, Wang ZB, Yuan XF, Wu SQ. 2013. Molecular biological identification of *Dermacentor nuttalli* and *Dermacentor marginatus*. *China Animal Husbandry and Veterinary Medicine* 40(9):8–14.

Ma HY. 2017. Discovery and identification of new viruses in ticks from northeastern China. Thesis, Jilin Agricultural University.

Moshaverinia A, Shayan P, Nabian S, Rahbari S. 2009. Genetic evidence for conspecificity between *Dermacentor marginatus* and *Dermacentor niveus*. [*Parasitology Research*](http://www.baidu.com/link?url=l2f1ekYBr6-LljXdIqbS--OEzEXQaTcCnYpLWQRW7CWvmPc0oUYE2u_Q0VwETkEPEJ0gHH6sPLNDHInnvE3Z86ezuhK_2wE38x7M_RTcviZsxpQuhAgHL5si3CKPPlw6ZjGLuAHQ5BPqdSgfo1GEEADHNVaCyPPuxlhfn__hWcm4NKVjgCLsIFI4LDqIhSAAtiyOMhcivVPRDpFts4VEQq3GOa42M6wwNnF53QX72pDzkNW3L8KSxShPoEog2a_ypdPPU2bPFZ798ODmvzs8o_) 105:1125–1132.

Rar VA, Livanova NN, Panov VV, Doroschenko EK, [Pukhovskaya](http://xueshu.baidu.com/s?wd=author:(Natalya M. Pukhovskaya) &tn=SE_baiduxueshu_c1gjeupa&ie=utf-8&sc_f_para=sc_hilight=person) NM, Vysochina NP, Ivanov LI. 2010. Genetic diversity of *Anaplasma* and *Ehrlichia* in the Asian part of Russia. *Ticks and Tick-borne Diseases* 1:57–65.

Selmi M, Tomassone L, Ceballos LA, Crisci A, Ragagli C, Pintore MD, Mignone W, Pautasso A, Ballardini M, Casalone C, Mannelli A. 2018. Analysis of the environmental and host-related factors affecting the distribution of the tick *Dermacentor marginatus*. *Experimental & Applied Acarolog* 75:1–17.

Shi ZY, Yang YS, Li Q, Zhao HB. 2004. Studies on species and geographic distribution of ticks in Gansu Province. *Chinese Journal of Veterinary Science and Technology* 34:48–49.

Spitalská [E](http://xueshu.baidu.com/s?wd=author:(Spitalská E) &tn=SE_baiduxueshu_c1gjeupa&ie=utf-8&sc_f_para=sc_hilight=person), [Stefanidesová](http://xueshu.baidu.com/s?wd=author:(Stefanidesová K) &tn=SE_baiduxueshu_c1gjeupa&ie=utf-8&sc_f_para=sc_hilight=person) K, [Kocianová](http://xueshu.baidu.com/s?wd=author:(Kocianová E) &tn=SE_baiduxueshu_c1gjeupa&ie=utf-8&sc_f_para=sc_hilight=person) E, [Boldiš](http://xueshu.baidu.com/s?wd=author:(Boldiš V) &tn=SE_baiduxueshu_c1gjeupa&ie=utf-8&sc_f_para=sc_hilight=person) V. 2012. *Rickettsia slovaca* and *Rickettsia raoultii* in *Dermacentor marginatus* and *Dermacentor reticulatus* ticks from Slovak Republic. *Experimental & Applied Acarology* 57:189–197.

Sun Y, Zheng SG, Xu RM. 2017. Systematic classification and pictorial key of *Dermacentor* ticks (Ixodida: Ixodidae) in China. *Acta Parasitologica ET Medica Entomologica Sinica* 24:25–40.

Teng GF, Jiang ZJ. 1991. *Economic Insect Fauna of China, Fasc 39, Acari: Ixodidae.* Beijing: Science Press.

Teng LJ. 2014. Investigation of three provinces in Northeast China the surface of cattle tick species and SFTSV nucleic acid detection. Thesis, Jilin Agricultural University.

Wang F, Liu GP, Ren QM, Liu Y, Cai ZL, Yin ZW, Sun W. 2005. Investigation of ticks in Dunhua, Jilin Province. *Chinese Journal of hygienic insecticides & Equipment* 11:321–322.

Wang JJ, Guo W, Liu J, Zhang ZB. 2014. Ticks checklist and tick-borne diseases in Inner Mongolia. *Acta Parasitologica et Medica Entomologica Sinica* 21(1):54–58.

Wang Z. 2014. The Identification and Evolutionary Analysis of Portion *Dermacentor* Species, and Serology Detected its Carrying *Babesiosis* in Xinjiang. *Master's thesis of Xinjiang Agricultural University.*

Wang Z. 2016. Investigation on the animal parasitic ticks and tick-borne zoonotic disease carrying situation in western of Liaoning Province. Thesis, Jinzhou Medical University.

Wei AM, Hu LM, Wu YM, Li HB, Zhang ZQ, Cao WC, Zhao QM. 2004. Ehrlichia DNA in ticks found in some parts of Northeast China. *Journal of Preventive Medicine of Chinese People's Liberation Army* 22(6):430–433.

[Xia](http://xueshu.baidu.com/s?wd=author:(Xia H) &tn=SE_baiduxueshu_c1gjeupa&ie=utf-8&sc_f_para=sc_hilight=person) H, [Hu C](http://xueshu.baidu.com/s?wd=author:(Hu C) &tn=SE_baiduxueshu_c1gjeupa&ie=utf-8&sc_f_para=sc_hilight=person), [Zhang D](http://xueshu.baidu.com/s?wd=author:(Zhang D) &tn=SE_baiduxueshu_c1gjeupa&ie=utf-8&sc_f_para=sc_hilight=person), [Tang](http://xueshu.baidu.com/s?wd=author:(Tang S) &tn=SE_baiduxueshu_c1gjeupa&ie=utf-8&sc_f_para=sc_hilight=person) S, [Zhang Z, Zheng K, Fan ZJ, Bente D, Zeng CQ, Li TX. 2015.](http://xueshu.baidu.com/s?wd=author:(Zhang Z) &tn=SE_baiduxueshu_c1gjeupa&ie=utf-8&sc_f_para=sc_hilight=person) Metagenomic profile of the viral communities in *Rhipicephalus* spp. Ticks from Yunnan, China. *PLOS ONE* 10:e0121609.

Yang JF, Tian HL, Guo RP, Zhang XG, Xu XT. 2015. Identification of tick species and epidemiological survey of ovine *theileria* parasite infection in Shangdong Province. *Chinese Journal of Veterinary Science* 35:930–937.

Yang LW, Guo XM, Li GJ, Hao S, Yang J, Xu WX, Wang FS, Li ZJ, Li YJ, Cui ZQ, Zhao XL, Hu MX, Cui YM. 2006. Investigation of ticks at 11 border ports of Heilongjiang Province. *Chinese Frontier Health Quarantine* 29:295–297.

Yang YS, Cao J, Zhao HB, Zhang JJ, DiWu JX, Gao XL. 2008a. Investigation on species and nature geographic distribution of ticks in Shaanxi Province. *Chinese Journal of Hygienic Insecticides and Equipments* 14:97–99.

Yang YS, DiWu JX, Cao J, Zhang JJ, Luo XH, Gao XL, Li Q, Zhang JM. 2008b. Investigation on kinds and nature geographic distribution of ticks in Qinghai Province. *Chinese Journal of Hygienic Insecticides and Equipments* 14:201–203.

Zhang F, Liu W, Wu XM, Xin ZT, Zhao QM, Yang H, Cao WC. 2008. Detection of *Francisella tularensis* in ticks and identification of their genotypes using multiple-locus variable-number tandem repeat analysis. *BMC Microbiology* 8:152.

Zhang GL, Zheng Z, Sun X, Liu XM, Liu R. Li HL. 2016. A survey of tick species and its distribution with the landscape structure in Xinjiang. *Chinese Journal of Vector Biology and Control* 27(5):432–435.

Zhang L, Li YX, Zhang K, Du JY, Chen CF, Wang KS, Wang YZ. 2014. Morphology and 16S r DNA Sequence Analysis of Ixodid Ticks Collected from Boertonggu pasture in Shihezi-Shawan region, Xinjiang. *Journal of Shihezi University (Natural Science)* 32(3):296–301.

Zhang YZ, Deng CY, Hu XB, Li JC, Liang D. 2009. Species and geographical distribution of ticks from Tibet. *Chinese Journal of Hygienic Insecticides and Equipments* 15:244–245.
