# Supplementary material for: Species delimitation of the Dermacentor ticks based on phylogenetic clustering and niche modeling
Source: PeerJ. 2019 May 10;7:e6911. doi: 10.7717/peerj.6911 (PMC6512763; doi:10.7717/peerj.6911)
Supplement: Table S3 [file peerj-07-6911-s006.docx]

Table S3. Correlation analysis of environmental variables

| 1 | 1.000 |  |  |  |  |  |  |  |  |  |  |  |  |  |  |  |  |  |  |
| --- | --- | --- | --- | --- | --- | --- | --- | --- | --- | --- | --- | --- | --- | --- | --- | --- | --- | --- | --- |
| 2 | -.416 | 1.000 |  |  |  |  |  |  |  |  |  |  |  |  |  |  |  |  |  |
| 3 | .601 | -.050 | 1.000 |  |  |  |  |  |  |  |  |  |  |  |  |  |  |  |  |
| 4 | -.735 | .570 | **-.817** | 1.000 |  |  |  |  |  |  |  |  |  |  |  |  |  |  |  |
| 5 | .600 | .220 | .067 | .051 | 1.000 |  |  |  |  |  |  |  |  |  |  |  |  |  |  |
| 6 | **.915** | -.612 | .714 | **-.929** | .271 | 1.000 |  |  |  |  |  |  |  |  |  |  |  |  |  |
| 7 | -.717 | .716 | -.712 | .980 | .101 | -.930 | 1.000 |  |  |  |  |  |  |  |  |  |  |  |  |
| 8 | -.285 | .246 | -.590 | .613 | .139 | -.507 | .577 | 1.000 |  |  |  |  |  |  |  |  |  |  |  |
| 9 | **.852** | -.435 | .700 | **-.827** | .366 | **.908** | -.799 | -.671 | 1.000 |  |  |  |  |  |  |  |  |  |  |
| 10 | .678 | .002 | -.002 | -.002 | **.948** | .347 | .003 | .229 | .371 | 1.000 |  |  |  |  |  |  |  |  |  |
| 11 | **.938** | -.529 | .752 | **-.924** | .317 | **.991** | **-.904** | -.482 | **.908** | .385 | 1.000 |  |  |  |  |  |  |  |  |
| 12 | .265 | -.628 | .239 | -.516 | -.298 | .451 | -.579 | -.344 | .335 | -.168 | .412 | 1.000 |  |  |  |  |  |  |  |
| 13 | -.085 | -.137 | -.222 | .123 | -.138 | -.127 | .078 | .202 | -.194 | .009 | -.112 | .595 | 1.000 |  |  |  |  |  |  |
| 14 | .138 | -.588 | .261 | -.508 | -.419 | .404 | -.577 | -.371 | .264 | -.355 | .332 | .715 | .021 | 1.000 |  |  |  |  |  |
| 15 | -.427 | .628 | -.420 | .681 | .127 | -.653 | .723 | .508 | -.581 | .114 | -.586 | -.499 | .327 | -.799 | 1.000 |  |  |  |  |
| 16 | -.019 | -.223 | -.133 | .003 | -.172 | -.024 | -.041 | .095 | -.099 | -.024 | -.014 | .698 | **.978** | .104 | .221 | 1.000 |  |  |  |
| 17 | .199 | -.622 | .309 | -.564 | -.392 | .465 | -.631 | -.422 | .346 | -.323 | .396 | .756 | .034 | **.985** | -.824 | .126 | 1.000 |  |  |
| 18 | -.491 | .020 | -.468 | .397 | -.425 | -.489 | .344 | .468 | -.624 | -.305 | -.489 | .359 | .753 | .142 | .311 | .699 | .095 | 1.000 |  |
| 19 | .616 | -.587 | .589 | -.761 | .050 | .778 | -.785 | -.684 | .771 | .093 | .741 | .690 | .090 | .534 | -.623 | .219 | .601 | -.385 | 1.000 |
|  | 1 | 2 | 3 | 4 | 5 | 6 | 7 | 8 | 9 | 10 | 11 | 12 | 13 | 14 | 15 | 16 | 17 | 18 | 19 |

**: significant at the 0.05 level *: significant at the 0.1 level

1. Bio1, Annual mean temperature; 2. Bio2, Mean diurnal range; 3. Bio3, Isothermality; 4. Bio4, Temperature seasonality; 5. Bio5, Max temperature of warmest month; 6. Bio6, Min temperature of coldest month; 7. Bio7, Annual temperature range; 8. Bio8, Mean temperature of wettest quarter; 9. Bio9, Mean temperature of driest quarter; 10. Bio10, Mean temperature of warmest quarter; 11. Bio11, Mean temperature of coldest quarter; 12. Bio12, Annual precipitation; 13. Bio13, Precipitation of wettest month; 14. Bio14, Precipitation of driest month; 15. Bio15, Precipitation seasonality; 16. Bio16, Precipitation of wettest quarter; 17. Bio17, Precipitation of driest quarter; 18. Bio18, Precipitation of warmest quarter; 19. Bio19, Precipitation of coldest quarter.
