# Supplementary material for: Species delimitation of the Dermacentor ticks based on phylogenetic clustering and niche modeling
Source: PeerJ. 2019 May 10;7:e6911. doi: 10.7717/peerj.6911 (PMC6512763; doi:10.7717/peerj.6911)
Supplement: Supplemental Information 1 [file peerj-07-6911-s008.docx]

MK208614

AATAAGAATTCTTATTCGAATAGAACTTAGCCAACCTGGGACATTAATTGGAAATGATCAAATTTATAACGTAATTGTTACTGCTCACGCTTTCATTATAATTTTTTTTATAGTTATACCTATTATAATCGGGGGGTTTGGAAATTGGCTTGTCCCAATTATATTAGGTGCTCCAGATATAGCTTTTCCCCGAATAAATAATATAAGGTTCTGATTACTTCCACCTTCACTATTCTTATTAATCAATTCTTCTTTAATTGAATCAGGAGCAGGGACTGGTTGAACAGTTTACCCTCCTTTATCTTCCAATTTATCACATTATGGCCCTTCAGTAGATTTAGCAATTTTCTCTCTTCATTTAGCAGGAGCATCATCAATTCTAGGAGCAATTAATTTTATTACTACAATTATTAACATACGATCAATTGGAATAACTCTTGAACGTATACCTTTATTTGTTTGATCAGTATTAATTACTGCAATTTTACTTTTACTTTCCTTACCTGTTTTAGCAGGGGCAATTACCATATTATTAACAGATCGAAATTTTAATACATCATTCTTTGACCCTTCAGGGGGGGGAGATCCAATTTTATATCAACATCTATTTTGATTTTTTGGTCAC

MK208615

AATAAGAATTCTTATTCGAATAGAACTTAGCCAACCTGGGACATTAATTGGAAATGATCAAATTTATAACGTAATTGTTACTGCTCACGCTTTCATTATAATTTTTTTTATAGTTATACCTATTATAATCGGGGGGTTTGGAAATTGGCTTGTCCCAATTATATTAGGTGCTCCAGATATAGCTTTTCCCCGAATAAATAATATAAGGTTCTGATTACTTCCACCTTCACTATTCTTATTAATCAATTCTTCTTTAATTGAATCAGGAGCAGGGACTGGTTGAACAGTTTACCCTCCTTTATCTTCCAATTTATCACATTATGGCCCTTCAGTAGATTTAGCAATTTTCTCTCTTCATTTAGCAGGAGCATCATCAATTCTAGGAGCAATTAATTTTATTACTACAATTATTAACATACGATCAATTGGAATAACTCTTGAACGTATACCTTTATTTGTTTGATCAGTATTAATTACTGCAATTTTACTTTTACTTTCCTTACCTGTTTTAGCAGGGGCAATTACCATATTATTAACAGATCGAAATTTTAATACATCATTCTTTGACCCTTCAGGGGGGGGAGATCCAATTTTATATCAACATCTATTCTGATTTTTTGGTCAC

MK208616

AATAAGAATTCTTATTCGAATAGAACTTAGCCAACCTGGGACATTAATTGGAAATGATCAAATTTATAACGTAATTGTTACTGCTCACGCTTTCATTATAATTTTTTTTATAGTTATACCTATTATAATCGGGGGGTTTGGAAATTGGCTTGTCCCAATTATATTAGGTGCTCCAGATATAGCTTTTCCCCGAATAAATAATATAAGGTTCTGATTACTTCCACCTTCACTATTCTTATTAATCAATTCTTCTTTAATTGAATCAGGAGCAGGGACTGGTTGAACAGTTTACCCTCCTTTATCTTCCAATTTATCACATTATGGCCCTTCAGTAGATTTAGCAATTTTCTCTCTTCATTTAGCAGGAGCATCATCAATTCTAGGAGCAATTAATTTTATTACTACAATTATTAACATACGATCAATTGGAATAACTCTTGAACGTATACCTTTATTTGTTTGATCAGTATTAATTACTGCAATTTTACTTTTACTTTCCTTACCTGTTTTAGCAGGGGCAATTACCATATTATTAACAGATCGAAATTTTAATACATCATTCTTTGACCCTTCAGGGGGGGGAGATCCAATTTTATATCAACATCTATTTTGATTTTTTGGTCAC

MK208617

AATAAGAATTCTTATTCGAATAGAACTTAGCCAACCTGGGACATTAATTGGAAATGATCAAATTTATAACGTAATTGTTACTGCTCACGCTTTCATTATAATTTTTTTTATAGTTATACCTATTATAATCGGGGGGTTTGGAAATTGGCTTGTCCCAATTATATTAGGTGCTCCAGATATAGCTTTTCCCCGAATAAATAATATAAGGTTCTGATTACTTCCACCTTCACTATTCTTATTAATCAATTCTTCTTTAATTGAATCAGGAGCAGGGACTGGTTGAACAGTTTACCCTCCTTTATCTTCCAATTTATCACATTATGGCCCTTCAGTAGATTTAGCAATTTTCTCTCTTCATTTAGCAGGAGCATCATCAATTCTAGGAGCAATTAATTTTATTACTACAATTATTAACATACGATCAATTGGAATAACTCTTGAACGTATACCTTTATTTGTTTGATCAGTATTAATTACTGCAATTTTACTTTTACTTTCCTTACCTGTTTTAGCAGGGGCAATTACCATATTATTAACAGATCGAAATTTTAATACATCATTCTTTGACCCTTCAGGGGGGGGAGATCCAATTTTATATCAACATCTATTTTGATTTTTTGGTCAC

MK208618

AATAAGAATTCTTATTCGAATAGAACTTAGCCAACCTGGGACATTAATTGGAAATGATCAAATTTATAACGTAATTGTTACTGCTCACGCTTTCATTATAATTTTTTTTATAGTTATACCTATTATAATCGGGGGGTTCGGAAATTGGCTTGTCCCAATTATATTAGGTGCTCCAGATATAGCTTTTCCCCGAATAAATAATATAAGATTCTGGTTACTTCCACCTTCACTATTCTTATTAATCAATTCTTCTTTAATTGAATCAGGAGCAGGGACTGGTTGAACAGTTTACCCTCCTTTATCTTCCAATTTATCACATTATGGCCCTTCAGTAGATTTAGCAATTTTCTCTCTTCATTTAGCAGGGGCATCATCAATTCTAGGAGCAATTAATTTCATTACTACAATTATTAACATACGATCAATTGGAATAACTCTTGAACGTATACCTTTATTTGTTTGATCAGTATTAATTACTGCAATTTTACTTTTACTTTCCTTACCTGTTTTAGCAGGAGCAATTACCATATTATTAACAGATCGAAATTTTAATACATCATTCTTTGACCCTTCAGGGGGGGGAGATCCAATTTTATATCAACATCTATTTTGATTTTTTGGTCAT

MK208619

AATAAGAATTCTTATTCGAATAGAACTTAGCCAACCTGGGACATTAATTGGAAATGATCAAATTTATAACGTAATTGTTACTGCTCACGCTTTCATTATAATTTTTTTTATAGTTATACCTATTATAATCGGGGGGTTCGGAAATTGGCTTGTCCCGATTATATTAGGTGCTCCAGATATAGCTTTTCCCCGAATAAATAATATAAGATTCTGGTTACTTCCACCTTCACTACTCTTATTAATCAATTCTTCTTTAATTGAATCAGGAGCAGGGACTGGTTGAACAGTTTACCCTCCTTTATCTTCCAATTTATCACATTATGGCCCTTCAGTAGATTTAGCAATTTTCTCTCTTCATTTAGCAGGGGCATCATCAATTCTAGGAGCAATTAATTTCATTACTACAATTATTAACATACGATCAATTGGAATAACTCTTGAACGTATACCTTTATTTGTTTGATCAGTATTAATTACTGCAATTTTACTTTTACTTTCCTTACCTGTTTTAGCAGGAGCAATTACCATATTATTAACAGATCGAAATTTTAATACATCATTCTTTGACCCTTCGGGGGGGGGAGATCCAATTTTATATCAACATCTATTTTGATTTTTTGGTCAC

MK208620

AATAAGAATTCTTATTCGAATAGAACTTAGCCAACCTGGGACATTAATTGGAAATGATCAAATTTATAACGTAATTGTTACTGCTCACGCTTTCATTATAATTTTTTTTATAGTTATACCTATTATAATCGGAGGGTTCGGAAATTGGCTTGTCCCGATTATATTAGGTGCTCCAGATATAGCTTTTCCCCGAATAAATAATATAAGATTCTGGTTACTTCCACCTTCACTACTCTTATTAATCAATTCTTCTTTAATTGAATCAGGAGCAGGGACTGGTTGAACAGTTTACCCTCCTTTATCTTCCAATTTATCACATTATGGCCCTTCAGTAGATTTAGCAATTTTCTCTCTTCATTTAGCAGGGGCATCATCAATTCTAGGAGCAATTAATTTCATTACTACAATTATTAACATACGATCAATTGGAATAACTCTTGAACGTATACCTTTATTTGTTTGATCAGTATTAATTACTGCAATTTTACTTTTACTTTCCTTACCTGTTTTAGCAGGAGCAATTACCATATTATTAACAGATCGAAATTTTAATACATCATTCTTTGACCCTTCAGGGGGGGGAGATCCAATTTTATATCAACATCTATTTTGATTTTTTGGTCCT

MK208621

AATAAGAATTCTTATTCGAATAGAACTTAGCCAACCTGGGACATTAATTGGAAATGATCAAATTTATAACGTAATTGTTACTGCTCACGCTTTCATTATAATTTTTTTTATAGTTATACCTATTATAATCGGGGGGTTCGGAAATTGGCTTGTCCCGATTATATTAGGTGCTCCAGATATAGCTTTTCCCCGAATAAATAATATAAGATTCTGGTTACTTCCACCTTCACTACTCTTATTAATCAATTCTTCTTTAATTGAATCAGGAGCAGGGACTGGTTGAACAGTTTACCCTCCTTTATCTTCCAATTTATCACATTATGGCCCTTCAGTAGATTTAGCAATTTTCTCTCTTCATTTAGCAGGGGCATCATCAATTCTAGGAGCAATTAATTTCATTACTACAATTATTAACATACGATCAATTGGAATAACTCTTGAACGTATACCTTTATTTGTTTGATCAGTATTAATTACTGCAATTTTACTTTTACTTTCCTTACCTGTTTTAGCAGGAGCAATTACCATATTATTAACAGATCGAAATTTTAATACATCATTCTTTGACCCTTCAGGGGGGGGAGATCCAATTTTATATCAACATCTATTTTGATTTTTTGGTCAC

MK208622

AATAAGAATTCTTATTCGAATAGAACTTAGCCAACCTGGGACATTAATTGGAAATGATCAAATTTATAACGTAATTGTTACTGCTCACGCTTTCATTATAATTTTTTTTATAGTTATACCTATTATAATCGGGGGGTTCGGAAATTGGCTTGTCCCGATTATATTAGGTGCTCCAGATATAGCTTTTCCCCGAATAAATAATATAAGATTCTGGTTACTTCCACCTTCACTACTCTTATTAATCAATTCTTCTTTAATTGAATCAGGAGCAGGGACTGGTTGAACAGTTTACCCTCCTTTATCTTCCAATTTATCACATTATGGCCCTTCAGTAGATTTAGCAATTTTCTCTCTTCATTTAGCAGGGGCATCATCAATTCTAGGAGCAATTAATTTCATTACTACAATTATTAACATACGATCAATTGGAATAACTCTTGAACGTATACCTTTATTTGTTTGATCAGTATTAATTACTGCAATTTTACTTTTACTTTCCTTACCTGTTTTAGCAGGAGCAATTACCATATTATTAACAGATCGAAATTTTAATACATCATTCTTTGACCCTTCAGGGGGGGGAGATCCAATTTTATATCAACATCTATTTTGATTTTTTTGGTCA

MK208623

AATAAGAATTCTTATTCGAATAGAACTTAGCCAACCTGGGACATTAATTGGAAATGATCAAATTTATAACGTAATTGTTACTGCTCACGCTTTCATTATAATTTTTTTTATAGTTATACCTATTATAATCGGGGGGTTCGGAAATTGGCTTGTCCCGATTATATTAGGTGCTCCAGATATAGCTTTTCCCCGAATAAATAATATAAGATTCTGGTTACTTCCACCTTCACTACTCTTATTAATCAATTCTTCTTTAATTGAATCAGGAGCAGGGACTGGTTGAACAGTTTACCCTCCTTTATCTTCCAATTTATCACATTATGGCCCTTCAGTAGATTTAGCAATTTTCTCTCTTCATTTAGCAGGGGCATCATCAATTCTAGGAGCAATTAATTTCATTACTACAATTATTAACATACGATCAATTGGAATAACTCTTGAACGTATACCTTTATTTGTTTGATCAGTATTAATTACTGCAATTTTACTTTTACTTTCCTTACCTGTTTTAGCAGGAGCAATTACCATATTATTAACAGATCGAAATTTTAATACATCATTCTTTGACCCTTCAGGGGGGGGAGATCCAATTTTATATCAACATCTATTTTGATTTTTTGGTCAC

MK208624

AATAAGAATTCTTATTCGAATAGAACTTAGCCAACCTGGGACATTAATTGGAAATGATCAAATTTATAACGTAATTGTTACTGCTCACGCTTTCATTATAATTTTTTTTATAGTTATACCTATTATAATCGGGGGGTTCGGAAATTGGCTTGTCCCGATTATATTAGGTGCTCCAGATATAGCTTTTCCCCGAATAAATAATATAAGATTCTGGTTACTTCCACCTTCACTACTCTTATTAATCAATTCTTCTTTAATTGAATCAGGAGCAGGGACTGGTTGAACAGTTTACCCTCCTTTATCTTCCAATTTATCACATTATGGCCCTTCAGTAGATTTAGCAATTTTCTCTCTTCATTTAGCAGGGGCATCATCAATTCTAGGAGCAATTAATTTCATTACTACAATTATTAACATACGATCAATTGGAATAACTCTTGAACGTATACCTTTATTTGTTTGATCAGTATTAATTACTGCAATTTTACTTTTACTTTCCTTACCTGTTTTAGCAGGAGCAATTACCATATTATTAACAGATCGAAATTTTAATACATCATTCTTTGACCCTTCGGGGGGGGGAGATCCAATTTTATATCAACATCTATTTTGATTTTTTGGTCAC

MK225574

GATAAGATTTTAATTCGAATAGAACTAGGGCAACCTGGTACATTAATTGGAAATGACCAAATCTATAATGTAATCGTTACTGCCCATGCTTTTATCATAATTTTTTTTATAGTTATACCAATTATAATTGGGGGATTTGGAAATTGATTAGTACCATTAATATTAGGCGCTCCTGACATAGCATTTCCTCGAATAAATAATATAAGATTCTGACTATTACCTCCCTCTTTATTCTTATTAATTAATTCATCTTTAGTTGAAAGAGGGGCGGGTACAGGGTGAACTGTATATCCTCCATTATCATCTAATTTATCCCATTATGGACCCTCAGTAGACATAGCAATTTTTTCACTCCACTTAGCAGGAGCCTCATCAATTTTAGGGGCTATTAATTTCATTACAACTATCATTAATATACGATCTTTAGGAATAACTTTAGAACGAATACCGCTATTTGTTTGATCAGTTCTAATTACCGCAATTCTCCTTCTGCTATCCTTACCTGTTCTTGCAGGTGCAATTACCATACTCTTGACAGATCGAAATTTTAACACCTCATTCTTTGACCCCTCAGGAGGAGGAGACCCAATTTTATATCAACACTTATTTTGATTTTTTGGACAC
