# Supplementary material for: Species delimitation of the Dermacentor ticks based on phylogenetic clustering and niche modeling
Source: PeerJ. 2019 May 10;7:e6911. doi: 10.7717/peerj.6911 (PMC6512763; doi:10.7717/peerj.6911)
Supplement: Supplemental Information 2 [file peerj-07-6911-s009.docx]

MK183100

CATATCAAGAGAGCCTTCGGCGCACAAGGGAACGTGCGTCCGTCGACTCGTTTTGACCGCGTCGGCATCACGGACAGTACGTTGAGTGCTGAAGCCACGCGCCAGCGGCCTCACGTGAGGGAGACGGTGGCGAACTGAACTGTTGTGCCAAAACTTCGCAGAGACGGAAACGAGGCATTGTACTACTGCAGCGCGACTAGTGCGCGCCTCCGAAGAGACCGCCGCAGGATGGAGTCGGACACCTGCAGGGAAAGTGCGGTCTGAATCGCGAGGCGCAAACGTCTGTTGCGACAGCAGTAGCGCGCACGTTTGCGAGAGAGTCGGAAGCTTGCGTGCACGCACAAATGCGGGAAGCAAATGCCGGCCGATTCCCGCGCCGTGCGCTAAGCGAGCGTGATCGCACGTTGCGTYGTTTGCCTTCGGAGTACGTCGAGCTCTTGCAGAAAAGGTCGCTCG

TCCGCGTCACCGCACGGGTGCAGGCGCCCTAGTCTGGGTTTCGTCGCAGGAATAGGAATCGGAAAAGATTCTTGCGCGGAACGGGGAGGACAAGGGTGCGCCCCGAAGCGGTAGTGACGCGGTTACGCGAGTGCGCCGTCTGCGAGCGCGAAGAAAACTGCACGACGGAAWAGTCGCCGCGAAGCGGAAAATGTCTCCTTCGAAAGCGTGGGTTTGCCCGTTGGCGGGGCTGAAGCGTTCCGTCGTAGTCCGCCGTCGGTCCAAGTGCTTCGCAGTCTCTGCCCCGAAAAGACTGGGCCACTCCAGTTGGGGCAGGGGCGACGCCACACGAGACGATGCCCTCTGCCAGGCTACGAGTCCGTCCTGCGGCGCCCGCTGAAGCGGCGCGCTGCGTGGGCGGCATGCCTCGGCGGTGTTTTGGGCTTCAGACACGGTCGTTTGCCACGCAACTGCTCGTGCGCCGCACGCGCGCAGGCGGTTTGTAAACCGCCTGCCAGCCTCGTCTATAAGTAGCTCCGTGTTGGGCGAAGGACGTGGTAGGGCGTCGCACTCGGTTCGCGCTGGGCTTTCGAACGTGTCGAGT

MK183101

CATATCAAGAGAGCCTTCGGCGCACAAGGGAACGTGCGTCCGTCGACTCGTTTTGACCGCGTCGGCATCACGGACAGTACGTTGAGTGCTGAAGCCACGCGCCAGCGGCCTCACGTGAGGGAGACGGTGGCGAACTGAACTGTTGTGCCAAAACTTCGCAGAGACGGAAACGAGGCATTGTACTACTGCAGCGCGACTAGTGCGCGCCTCCGAAGAGACCGCCGCAGGATGGAGTCGGACACCTGCAGGGAAAGTGTGGTCTGAATCGCGAGGCGCAAACGTCTGTTGCGACAGCAGTAGCGCGCACGTTTGCGAGAGAGTCGGAAGCTTGCGTGCACGCACAAATGCGGGAAGCAAATGCCGGCCGATTCCCGCGCCGTGCGCTAAGCGAGCGTGATCGCACGTTGCGTTGTTTGCCTTCGGAGTACGTCGAGCTCTTGCAGAAAAGGTCGCTCG

TCCGCGTCACCGCACGGGTGCAGGCGCCCTAGTCTGGGTTTCGTCGCAGGAATAGGAATCGGAAAAGATTCTTGCGCGGAACGGGGAGGACAAGGGTGCGCCCCGAAGCGGTAGTGACGCGGTTACGCGAGTGCGCCGTCTGCGAGCGCGAAGAAAACTGCACGACGGAAWAGTCGCCGCGAAGCGGAAAATGTCTCCTTCGAAAGCGTGGGTTTGCCCGTTGGCGGGGCTGAAGCGTTCCGTCGTAGTCCGCCGTCGGTCCAAGTGCTTCGCAGTCTCTGCCCCGAAAAGACTGGGCCACTCCAGTTGGGGCAGGGGCGACGCCACACGAGACGATGCCCTCTGCCAGGCTACGAGTCCGTCCTGCGGCGCCCGCTGAAGCGGCGCGCTGCGTGGGCGGCATGCCTCGGCGGTGTTTTGGGCTTCAGACACGGTCGTTTGCCACGCAACTGCTCGTGCGCCGCACGCGCGCAGGCGGTTTGTAAACCGCCTGCCAGCCTCGTCTATAAGTAGCTCCGTGTTGGGCGAAGGACGTGGTAGGGCGTCGCACTCGGTTCGCGCTGGGCTTTCGAACGTGTCGAGT

MK183102

CATATCAAGAGAGCCTTCGGCGCACAAGGGAACGTGCGTCCGTCGACTCGTTTTGACCGCGTCGGCATCACGGACAGTACGTTGAGTGCTGAAGCCACGCGCCAGCGGCCTCACGTGAGGGAGACGGTGGCGAACTGAACTGTTGTGCCAAAACTTCGCAGAGACGGAAACGAGGCATTGTACTACTGCAGCGCGACTAGTGCGCGCCTCCGAAGAGACCGCCGCAGGATGGAGTCGGACACCTGCAGGGAAAGTGCGGTCTGAATCGCGAGGCGCAAACGTCTGTTGCGACAGCAGTAGCGCGCACGTTTGCGAGAGAGTCGGAAGCTTGCGTGCACGCACAAATGCGGGAAGCAAATGCCGGCCGATTCCCGCGCCGTGCGCTAAGCGAGCGTGATCGCACGTTGCGTCGTTTGCCTTCGGAGTACGTCGAGCTCTTGCAGAAAAGGTCGCTCG

TCCGCGTCACCGCACGGGTGCAGGCGCCCTAGTCTGGGTTTCGTCGCAGGAATAGGAATCGGAAAAGATTCTTGCGCGGAACGGGGAGGACAAGGGTGCGCCCCGAAGCGGTAGTGACGCGGTTACGCGAGTGCGCCGTCTGCGAGCGCGAAGAAAACTGCACGACGGAAAAGTCGCCGCGAAGCGGAAAATGTCTCCTTCGAAAGCGTGGGTTTGCCCGTTGGCGGGGCTGAAGCGTTCCGTCGTAGTCCGCCGTCGGTCCAAGTGCTTCGCAGTCTCTGCCCCGAAAAGACTGGGCCACTCCAGTTGGGGCAGGGGCGACGCCACACGAGACGATGCCCTCTGCCAGGCTACGAGTCCGTCCTGCGGCGCCCGCTGAAGCGGCGCGCTGCGTGGGCGGCATGCCTCGGCGGTGTTTTGGGCTTCAGACACGGTCGTTTGCCACGCAACTGCTCGTGCGCCGCACGCGCGCAGGCGGTTTGTAAACCGCCTGCCAGCCTCGTCTATAAGTAGCTCCGTGTTGGGCGAAGGACGTGGTAGGGCGTCGCACTCGGTTCGCGCTGGGCTTTCGAACGTGTCGAGT

MK183103

CATATCAAGAGAGCCTTCGGCGCACAAGGGAACGTGCGTCCGTCGACTCGTTTTGACCGCGTCGGCATCACGGACAGTACGTTGAGTGCTGAAGCCACGCGCCAGCGGCCTCACGTGAGGGAGACGGTGGCGAACTGAACTGTTGTGCCAAAACTTCGCAGAGACGGAAACGAGGCATTGTACTACTGCAGCGCGGCTAGTGCGCGCCTCCGAAGAGACCGCCGCAGGATGGAGTCGGACACCTGCAGGGAAAGTGCGGTCTGAATCGCGAGGCGCAAACGTCTGTTGCGACAGCAGTAGCGCGCACGTTTGCGAGAGAGTCGGAAGCTTGCGTGCACGGACAAACGCGGGAAGCAAATGCCGGCCGATTCCCGCGCCGTGCGCTAAGCGAGCGTGATCGCACGTTGCGTTGTTTGCCTTCGGAGTACGTCGAGCTCTTGCAAAAAGGTCACTCG

TCCGCGTCACCGCACGGGTGCAGGCGCCCTAGTCTGGGTTTCGTCGCAGGAATAGGAATCGGAAAAAATTCTTGCGCGGAACGGGGAGGACAAGGGTGCGCCCCGAAGCGGTAGTGATGCGGTTACGCGAGTGCGCCGTCTGCGAGCGCGAAGAAAACTGCACGACGAAATAGTCGCCGCGAAGCGGAAAATGTCTCCTTCGAAAGCGTGGGTTTGCCCGTTGGCGGGGCTGAAGCGTTCCGTCGTAGTCCGCCGTCGGTCCAAGTGCTTCGCAGTCTCTGCCCCGAAAAGACTGGGCCACTCCAGTTGGGGCAGGGGCGACGCCACACGAGACGATGCCCTCTGCCAGGCTACGAGTCCGTCCTGCGGCGCCCGCTGAAGCGGCGCGCTGCGTGGGCGGCATGCCTCGGCGGTGTTTTGGGCTGCAGACACGGTCGTTTGCCACGCAACTGCTCGTGCGCCGCACGCGCGCAGGCGGTTTGTAAACCGCCTGCCAGCCTCGTCTATAAGTAGCTCCGTGTTGGGCGAAGGACGTGGTAGGGCGTCGCACTCGGTTCGCGCTGGGCTTTCGAACGTGTCGAGT

MK183104

CATATCAAGAGAGCCTTCGGCGCACAAGGGAACGTGCGTCCGTCGACTCGTTTTGACCGCGTCGGCATCACGGACAGTACGTTGAGTGCTGAAGCCACGCGCCAGCGGCCTCACGTGAGGGAGACGGTGGCGAACTGAACTGTTGTGCCAAAACTTCGCAGAGACGGAAACGAGGCATTGTACTACTGCAGCGCGGCTAGTGCGCGCCTCCGAAGAGACCGCCGCAGGATGGAGTCGGACACCTGCAGGGAAAGTGCGGTCTGAATCGCGAGGCGCAAACGTCTGTTGCGACAGCAGTAGCGCGCACGTTTGCGAGAGAGTCGGAAGCTTGCGTGCACGGACAAACGCGGGAAGCAAATGCCGGCCGATTCCCGCGCCGTGCGCTAAGCGAGCGTGATCGCACGTTGCGTTGTTTGCCTTCGGAGTACGTCGAGCTCTTGCAAAAAGGTCACTCGTCCGCGTCACCGCACGGGTGCAGGCGCCCTAGTCTGGGTTTCGTCGCAGGAATAGGAATCGGAAAAAATTCTTGCGCGGAACGGGGAGGACAAGGGTGCGCCCCGAAGCGGTAGTGATGCGGTTACGCGAGTGCGCCGTCTGCGAGCGCGAAGAAAACTGCACGACGGAATAGTCGCCGCGAAGCGGAAAATGTCTCCTTCGAAAGCGTGGGTTTGCCCGTTGGCGGGGCTGAAGCGTTCCGTCGTAGTCCGCCGTCGGTCCAAGTGCTTCGCAGTCTCTGCCCCGAAAAGACTGGGCCACTCCAGTTGGGGCAGGGGCGACGCCACACGAGACGATGCCCTCTGCCAGGCTACGAGTCCGTCCTGCGGCGCCCGCTGAAGCGGCGCGCTGCGTGGGCGGCATGCCTCGGCGGTGTTTTGGGCTGCAGACACGGTCGTTTGCCACGCAACTGCTCGTGCGCCGCACGCGCGCAGGCGGTTTGTAAACCGCCTGCCAGCCTCGTCTATAAGTAGCTCCGTGTTGGGCGAAGGACGTGGTAGGGCGTCGCACTCGGTTCGCGCTGGGCTTTCGAACGTGTCGAGT

MK183105

CATATCAAGAGAGCCTTCGGCGCACAAGGGAACGTGCGTCCGTCGACTCGTTTTGACCGCGTCGGCATCACGGACAGTACGTTGAGTGCTGAAGCCACGCGCCAGCGGCCTCACGTGAGGGAGACGGTGGCGAACTGAACTGTTGTGCCAAAACTTCGCAGAGACGGAAACGAGGCATTGTACTACTGCAGCGCGGCTAGTGCGCGCCTCCGAAGAGACCGCCGCAGGATGGAGTCGGACACCTGCAGGGAAAGTGCGGTCTGAATCGCGAGGCGCAAACGTCTGTTGCGACAGCAGTAGCGCGCACGTTTGCGAGAGAGTCGGAAGCTTGCGTGCACGGACAAACGCGGGAAGCAAATGCCGGCCGATTCCCGCGCCGTGCGCTAAGCGAGCGTGATCGCACGTTGCGTTGTTTGCCTTCGGAGTACGTCGAGCTCTTGCAAAAAGGTCACTCG

TCCGCGTCACCGCACGGGTGCAGGCGCCCTAGTCTGGGTTTCGTCGCAGGAATAGGAATCGGAAAAAATTCTTGCGCGGAACGGGGAGGACAAGGGTGCGCCCCGAAGCGGTAGTGATGCGGTTACGCGAGTGCGCCGTCTGCGAGCGCGAAGAAAACTGCACGACGGAATAGTCGCCGCGAAGCGGAAAATGTCTCCTTCGAAAGCGTGGGTTTGCCCGTTGGCGGGGCTGAAGCGTTCCGTCGTAGTCCGCCGTCGGTCCAAGTGCTTCGCAGTCTCTGCCCCGAAAAGACTGGGCCACTCCAGTTGGGGCAGGGGCGACGCCACACGAGACGATGCCCTCTGCCAGGCTACGAGTCCGTCCTGCGGCGCCCGCTGAAGCGGCGCGCTGCGTGGGCGGCATGCCTCGGCGGTGTTTTGGGCTGCAGACACGGTCGTTTGCCACGCAACTGCTCGTGCGCCGCACGCGCGCAGGCGGTTTGTAAACCGCCTGCCAGCCTCGTCTATAAGTAGCTCCGTGTTGGGCGAAGGACGTGGTAGGGCGTCGCACTCGGTTCGCGCTGGGCTTTCGAACGTGTCGAGT

MK183106

CATATCAAGAGAGCCTTCGGCGCACAAGGGAACGTGCGTCCGTCGACTCGTTTTGACCGCGTCGGCATCACGGACAGTACGTTGAGTGCTGAAGCCACGCGCCAGCGGCCTCACGTGAGGGAGACGGTGGCGAACTGAACTGTTGTGCCAAAACTTCGCAGAGACGGAAACGAGGCATTGTACTACTGCAGCGCGGCTAGTGCGCGCCTCCGAAGAGACCGCCGCAGGATGGAGTCGGACACCTGCAGGGAAAGTGCGGTCTGAATCGCGAGGCGCAAACGTCTGTTGCGACAGCAGTAGCGCGCACGTTTGCGAGAGAGTCGGAAGCTTGCGTGCACGGACAAACGCGGGAAGCAAATGCCGGCCGATTCCCGCGCCGTGCGCTAAGCGAGCGTGATCGCACGTTGCGTTGTTTGCCTTCGGAGTACGTCGAGCTCTTGCAAAAAGGTCACTCG

TCCGCGTCACCGCACGGGTGCAGGCGCCCTAGTCTGGGTTTCGTCGCAGGAATAGGAATCGGAAAAAATTCTTGCGCGGAACGGGGAGGACAAGGGTGCGCCCCGAAGCGGTAGTGATGCGGTTACGCGAGTGCGCCGTCTGCGAGCGCGAAGAAAACTGCACGACGGAATAGTCGCCGCGAAGCGGAAAATGTCTCCTTCGAAAGCGTGGGTTTGCCCGTTGGCGGGGCTGAAGCGTTCCGTCGTAGTCCGCCGTCGGTCCAAGTGCTTCGCAGTCTCTGCCCCGAAAAGACTGGGCCACTCCAGTTGGGGCAGGGGCGACGCCACACGAGACGATGCCCTCTGCCAGGCTACGAGTCCGTCCTGCGGCGCCCGCTGAAGCGGCGCGCTGCGTGGGCGGCATGCCTCGGCGGTGTTTTGGGCTGCAGACACGGTCGTTTGCCACGCAACTGCTCGTGCGCCGCACGCGCGCAGGCGGTTTGTAAACCGCCTGCCAGCCTCGTCTATAAGTAGCTCCGTGTTGGGCGAAGGACGTGGTAGGGCGTCGCACTCGGTTCGCGCTGGGCTTTCGAACGTGTCGAGT

MK183107

TATATCAAGAGAGTGTCGTGCGCCCGTTGGGTGCGGTGCTCGAGACTCGTTTTGACCGCGTCGGCGTTATGGACAGCACGTTGAACGTGAAAAGCTTGTGCAGAGGACGTCGTTGGACGAGGAGATGTCAGAGATTGGAGTGCACGCGCAAAGGGGAGGGTCCCGGGGGATGCGTTCCCCGGCCCTCCAGCGCTGGCGTGTTGGGAAGTCTGAATCGCGCGCGAAGCGGGATCGCCCTTCGAAAAGCGTCTGGAGTCGGAATCGGGACGCCGAATCGGATTCGAAGGTTGCGTGAGCGCGTGTGAAAGACGACGCCGGATGAGCGCTTGGCGGGGAACGGATGCATCCCCTGCGCTCGTGCCTCTTACGACACTTTCTCTCGGAAACCGCGAATCGGCTGCGAAACCTCCCGAAGGGTGCGGAGGCGAGCAGAAGCGGTGAGCCGCGAATCGGCTGCGAAACCTCCCGAAGGGTGCGGAGGCGAGCAGAAGCGGACGCCGAGAGTCCTGGCACACCGTCGGAAGAAGGGCTAAGTGCAGCCGAGCGCGCCTGAAGGTGCGCACGGCGTGCGGATCCTTTTTTTTCCCCTCGGCTTGTGCAGTCGTCTCTGAGATTCGGAATTCTCCGAGTTAGAGTCCACGACGAGGAGAGTTGAAACGCGAATCGGCCGCGGCAGTCCTTTCGATGGGTGAAAAGGGCGCGTGGAGGCGTTTCCTCTCCGTTTCGAACGCGCGCGCGTTGGCCCCCCGGGAATTAGGGCGCCCCGCCGGCTTCGACCAAACCTCGGCTGCCAGGAAAGCCCTCCGACCTGGGAGGAAGATGGCTGGTGTGTGTTGGGGAAGGAGTTTCTTGCCGTC
